# Supplementary material for: Support received after bereavement by suicide and other sudden deaths: a cross-sectional UK study of 3432 young bereaved adults
Source: BMJ Open. 2017 May 29;7(5):e014487. doi: 10.1136/bmjopen-2016-014487 (PMC5729987; doi:10.1136/bmjopen-2016-014487)
Supplement: Supplementary table [file bmjopen-2016-014487supp001.pdf]

Supplementary Table: Estimates of the relationship between support outcomes and bereavement exposure (suicide *versus* non-suicide sudden death)

**Exposure group**	**Non-suicide sudden death (n=2818)**		**Suicide (n = 614)**				
**Primary outcomes**	**Prevalence n (%)**	**Odds ratio** (reference)	**Prevalence n (%)**	**Unadjusted odds ratio (95% CI)**	***p* value***	**Adjusted[a] odds ratio (95% CI)**	***p* value***
receipt of formal support post-bereavement	1048 (37)	1	221 (36)	0.96 (0.79-1.16)	0.644	1.08 (0.88-1.31)	0.472
receipt of informal support post-bereavement	1887 (67)	1	389 (63)	0.82 (0.67-0.99)	0.042	**0.78†** **(0.64-0.96)**	**0.018**
**Secondary outcomes**							
no support post-bereavement [b]	557 (20)	1	141 (23)	1.31 (1.05-1.62)	0.016	1.32 (1.06-1.67)	0.014
immediate receipt of support (<1 week)	1219 (43)	1	219 (36)	**0.74** **(0.61-0.90)**	**0.002**	**0.74†** **(0.61-0.90)**	**0.003**
delayed receipt of valuable support (>6 months)	1105 (39)	1	271 (44)	1.24 (1.02-1.49)	0.028	1.28 (1.05-1.56)	0.017
use of formal support exclusively [c]	293/2131 (14)	1	68/441 (15)	1.18 (0.88-1.59)	0.264	1.27 (0.93-1.72)	0.130
help sought for post-bereavement self-harm [d]	50/154 (32)	1	19/56 (34)	1.02 (0.52-2.02)	0.949	1.22 (0.57-2.61)	0.612

[a] adjusted for age, gender, socio-economic status, pre-loss depression, pre-loss suicidal and non-suicidal self-harm, other family history of suicide (excluding index bereavement), time since bereavement, and kinship to the deceased.

[b] outcome excluded those who solely endorsed that they chose to handle the bereavement alone

[c] in sub-set of n=2,572 receiving support after bereavement

[d] in sub-set of n=210 who had attempted suicide post-bereavement

* significance threshold of p=0·05 for primary outcomes and p=0·01 for secondary outcomes

† association no longer significant when stigma added to final adjusted model
