# Supplementary material for: Support received after bereavement by suicide and other sudden deaths: a cross-sectional UK study of 3432 young bereaved adults
Source: BMJ Open. 2017 May 29;7(5):e014487. doi: 10.1136/bmjopen-2016-014487 (PMC5729987; doi:10.1136/bmjopen-2016-014487)
Supplement: Supplementary STROBE Checklist [file bmjopen-2016-014487supp002.docx]

**STROBE checklist for cross-sectional study of support received after bereavement by suicide and other sudden deaths (UCL Bereavement Study)**

**Corresponding author: Dr Alexandra Pitman 27 September 2016**

Checklist of items that should be included in reports of ***cross-sectional studies***: green denotes page number

<http://www.strobe-statement.org/index.php?id=available-checklists>

|  | Item No | Recommendation |
| --- | --- | --- |
| **Title and abstract**  **Page 1 & 2-3** | 1 | **(*a*) Indicate the study’s design with a commonly used term in the title or the abstract:** abstract/title indicates that we conducted a national cross-sectional study |
|  |  | **(*b*) Provide in the abstract an informative and balanced summary of what was done and what was found:** abstract outlines our hypothesis, exposures and outcomes, and adjusted odds ratio for the associations hypothesised |
| Introduction | | |
| Background/rationale  **Page 4** | 2 | **Explain the scientific background and rationale for the investigation being reported:** Our introduction outlines the policy context, including key research references, and highlights the lack of evidence to support current suicide prevention strategy. |
| Objectives  **Page 3 & 4** | 3 | **State specific objectives, including any prespecified hypotheses:**  Objectives and primary hypothesis stated in the Abstract and Introduction. Our objective was to conduct a population-based survey comparing the support received by people bereaved by different modes of sudden death and to test specific hypotheses regarding inequalities in support received by people bereaved by suicide. |
| Methods | | |
| Study design  **Page 5-6** | 4 | **Present key elements of study design early in the paper:** Cross-sectional survey stated in Methods. |
| Setting  **Page 5-6** | 5 | **Describe the setting, locations, and relevant dates, including periods of recruitment, exposure, follow-up, and data collection**: Describes emailing individuals at 37 HEIs in 2010 for cross-sectional data collection. Acknowledgement section details the locations of diverse participating HEIs. |
| Participants  **Page 5-6** | 6 | **Give the eligibility criteria, and the sources and methods of selection of participants:** Eligibility criteria described as: people aged 18-40 who had experienced sudden bereavement of a close friend or relative after ten years of age. |
| Variables  **Page 7-8** | 7 | **Clearly define all outcomes, exposures, predictors, potential confounders, and effect modifiers. Give diagnostic criteria, if applicable.**  All outcomes described, denoting how these were derived.  Exposure clearly defined. Eight pre-specified confounding variables defined and justified. Kinship defined as a potential effect modifier. |
| Data sources/ measurement  **Page 5 &7-8** | 8* | **For each variable of interest, give sources of data and details of methods of assessment (measurement). Describe comparability of assessment methods if there is more than one group:**  Questionnaire development and content described. Same instrument used for all exposure groups. |
| Bias  **Page 5-6** | 9 | **Describe any efforts to address potential sources of bias:** We describe how we followed-up non-responding HEIs to ensure a diverse representation of HEIs, and how we masked participants to the study hypothesis. We also describe a decision to use two-tailed analysis to reduce inductive bias. |
| Study size  **Page 6** | 10 | **Explain how the study size was arrived at:** We based our sample size calculation on the primary outcome of a separate study to describe the association between suicide bereavement and suicide attempt (the rarest outcome), to detect a doubling of the UK community prevalence of lifetime suicide attempt (6·5%) in young adult samples. |
| Quantitative variables  **Page 5-8** | 11 | **Explain how quantitative variables were handled in the analyses. If applicable, describe which groupings were chosen and why:** Our Methods section defines the 3 exposure groups, 7 outcomes, and 8 covariates; and how each was used in the analysis. |
| Statistical methods  **Page 8-9** | 12 | **(*a*) Describe all statistical methods, including those used to control for confounding:** We describe our use of multivariable logistic regression, including justification of the 8 covariates used in the adjusted models. |
|  |  | **(*b*) Describe any methods used to examine subgroups and interactions:** We describe how we tested for an interaction with kinship. |
|  |  | **(*c*) Explain how missing data were addressed:** We explain that levels of missing data were low (<7% for model covariates, and <4% for outcomes) and so our statistician co-authors advised that we did not need to use best and worst case scenarios to impute missing values as part of our sensitivity analyses. |
|  |  | **(*d*) If applicable, describe analytical methods taking account of sampling strategy:** We describe our use of a cluster variable to take into account the potential for clustering of responses at the HEI level. |
|  |  | **(*e*) Describe any sensitivity analyses:** We describe sensitivity analyses that assessed the impact of simulating more stringent inclusion criteria for the sampling strategy. |
| Results | | |
| Participants  **Page 9, Figure 1** | 13* | **(a) Report numbers of individuals at each stage of study—eg numbers potentially eligible, examined for eligibility, confirmed eligible, included in the study, completing follow-up, and analysed:** We specify numbers of those participating, consenting, and eligible, and present the participant flow in Figure 1. |
|  |  | **(b) Give reasons for non-participation at each stage:** numbers not consenting, not eligible, not indicating exposure group, and not providing at least 1 outcome measure presented in Figure 1. |
|  |  | **(c) Consider use of a flow diagram**: see Figure 1 |
| Descriptive data  **Page 9-10, Tables 1 & 2** | 14* | **(a) Give characteristics of study participants (eg demographic, clinical, social) and information on exposures and potential confounders:** Tables 1 & 2 and text indicates descriptive characteristics by exposure group. |
|  |  | **(b) Indicate number of participants with missing data for each variable of interest**: Tables 1 & 2 provide proportion of missing values for each covariate of interest by exposure group. |
| Outcome data  **Table 3** | 15* | **Report numbers of outcome events or summary measures:** Table 3 presents prevalence for each outcome by exposure group. |
| Main results  **Page 10-11, Tables 3 & 4** | 16 | **(*a*) Give unadjusted estimates and, if applicable, confounder-adjusted estimates and their precision (eg, 95% confidence interval). Make clear which confounders were adjusted for and why they were included:** Text and Tables 3 and 4 provide unadjusted and adjusted estimates, with 95% confidence intervals and p-values. |
|  |  | **(*b*) Report category boundaries when continuous variables were categorized**: N/A |
|  |  | **(*c*) If relevant, consider translating estimates of relative risk into absolute risk for a meaningful time period:** N/A |
| Other analyses  **Page 11** | 17 | **Report other analyses done—eg analyses of subgroups and interactions, and sensitivity analyses:** We report: results of adding stigma to final models, tests for an interaction with gender, sensitivity analyses. |
| Discussion | | |
| Key results  **Page 11-12** | 18 | **Summarise key results with reference to study objectives:** The start of our discussion summarises the principle findings in relation to our main hypothesis. |
| Limitations  **Page 12-13** | 19 | **Discuss limitations of the study, taking into account sources of potential bias or imprecision. Discuss both direction and magnitude of any potential bias:** Our discussion summarises both the strengths and weaknesses of this study, both in comparison with other potential approaches, and other previously-used approaches. We consider the possibility of either over- or under-estimation of risks given specific potential biases. |
| Interpretation  **Page 11-13** | 20 | **Give a cautious overall interpretation of results considering objectives, limitations, multiplicity of analyses, results from similar studies, and other relevant evidence:** Our discussion compares our findings to the existing literature and comments on the degree to which our findings are consistent with this, and the extent to which they contribute to policy developments in relation to provision of support after suicide bereavement. |
| Generalisability  **Page 12-13** | 21 | **Discuss the generalisability (external validity) of the study results:** We explore the degree to which findings from a primarily female and highly-educated UK HEI population are generalizable, either in the UK or internationally. |
| Other information | | |
| Funding  **Page 15** | 22 | **Give the source of funding and the role of the funders for the present study and, if applicable, for the original study on which the present article is based:** Our footnotes identify the MRC as the funder, and the limits of their role in this study. |

*Give information separately for exposed and unexposed groups.

**Note:** An Explanation and Elaboration article discusses each checklist item and gives methodological background and published examples of transparent reporting. The STROBE checklist is best used in conjunction with this article (freely available on the Web sites of PLoS Medicine at http://www.plosmedicine.org/, Annals of Internal Medicine at http://www.annals.org/, and Epidemiology at http://www.epidem.com/). Information on the STROBE Initiative is available at www.strobe-statement.org.
