# Supplementary material for: Support received after bereavement by suicide and other sudden deaths: a cross-sectional UK study of 3432 young bereaved adults
Source: BMJ Open. 2017 May 29;7(5):e014487. doi: 10.1136/bmjopen-2016-014487 (PMC5729987; doi:10.1136/bmjopen-2016-014487)
Supplement: Author's manuscript [file bmjopen-2016-014487.draft_revisions.pdf]

BMJ Open

Support received after bereavement by suicide and other sudden deaths: a cross-sectional UK study of 3432 bereaved adults

Journal:	*BMJ Open*
Manuscript ID	bmjopen-2016-014487
Article Type:	Research
Date Submitted by the Author:	27-Sep-2016
Complete List of Authors:	Pitman, Alexandra; UCL, UCL Division of Psychiatry<br>Rantell, Khadija; University College London, Institute of Neurology<br>Moran, Paul; University of Bristol<br>Sireling, Lester; independent medico-legal practice<br>Marston, Louise; UCL, Primary Care and Population Health<br>King, Michael; University College Medical School, UCL Division of Psychiatry<br>Osborn, David; UCL, UCL Division of Psychiatry
**Primary Subject Heading**:	Mental health
Secondary Subject Heading:	Epidemiology, Health services research, Mental health, Public health
Keywords:	MENTAL HEALTH, Suicide & self-harm < PSYCHIATRY, PUBLIC HEALTH, Organisation of health services < HEALTH SERVICES ADMINISTRATION & MANAGEMENT

SCHOLARONE™
Manuscripts

For peer review only

Support received after bereavement by suicide and other sudden deaths: a cross-sectional UK study of 3432 bereaved adults

Dr Alexandra L. Pitman, Honorary Research Associate, UCL Division of Psychiatry, University College London, Maple House, 149 Tottenham Court Rd, London W1T 7NF, UK (corresponding author) a.pitman@ucl.ac.uk

Dr Khadija Rantell, Research Associate, UCL Institute of Neurology, University College London, Queen Square, London WC1N 3BG, UK

Dr Paul Moran, Reader in Psychiatry, School of Social and Community Medicine, University of Bristol, Oakfield House, Oakfield Grove, Clifton, Bristol BS8 2BN, UK

Dr Lester Sireling, Consultant psychiatrist in independent medico-legal practice, c/o UCL Division of Psychiatry, University College London, Maple House, 149 Tottenham Court Rd, London W1T 7NF, UK

Dr Louise Marston, Senior Research Associate, UCL Research Department of Primary Care & Population Health, University College London, Rowland Hill Street, London NW3 2PF, UK

Professor Michael B. King, Professor of Primary Care Psychiatry, UCL Division of Psychiatry, University College London, Maple House, 149 Tottenham Court Rd, London W1T 7NF, UK

Professor David P.J. Osborn, Professor of Psychiatric Epidemiology, UCL Division of Psychiatry, University College London, Maple House, 149 Tottenham Court Rd, London W1T 7NF, UK

For peer review only - http://bmjopen.bmj.com/site/about/guidelines.xhtml

Abstract

Objective: To test the hypothesis that people bereaved by suicide are less likely to receive formal or informal support than people bereaved by other causes of sudden death.

Design: National cross-sectional study.

Setting: Adults working or studying at any UK Higher Education Institution (HEI) in 2010.

Participants: 3,432 eligible respondents aged 18-40 bereaved by the sudden death of a close friend or relative, sampled from approximately 659,572 bereaved and non-bereaved staff and students at 37 of 164 UK HEIs invited to participate.

Exposures: Bereavement by suicide (n=614; 18%), by sudden unnatural causes (n=712; 21%) and by sudden natural causes (n=2106; 61%).

Main outcome measures: Receipt of formal and of informal support post-bereavement; timing of valued support.

Results: 21% (725/3432) of our sample of bereaved adults reported having received no formal or informal bereavement support, with no evidence for group differences. People bereaved by suicide were less likely to have received informal support than those bereaved by sudden natural causes (adjusted odds ratio (AOR) = 0.79; 95% CI=0.64 to 0.98), or unnatural causes (AOR=0.74; 95% CI=0.58 to 0.96), but did not differ from either comparison group on receipt of formal support. People bereaved by suicide were less likely to have received immediate support (AOR=0.73; 95% CI=0.59 to 0.90) and more likely to report delayed receipt of support (AOR=1.33; 95% CI=1.08 to 1.64) than people bereaved by sudden natural causes. Associations were not modified by gender, and became non-significant when adding stigma to final models.

For peer review only - http://bmjopen.bmj.com/site/about/guidelines.xhtml

Conclusions: People bereaved by suicide are less likely to receive informal support than people bereaved by other causes of sudden death, and are more likely to perceive delays in accessing any support. This is concerning given their higher risk of suicide attempt and the recommendations within suicide prevention strategies regarding their need for support.

Study registration: http://www.ucl.ac.uk/psychiatry/bereavementstudy/

Strengths and limitations of this study

- We conducted a large population-based closed survey to identify bereaved friends and relatives, avoiding the biases inherent to using a help-seeking sample.
- We captured use of a wide range of formal and informal support sources, and the time taken to access valued support.
- We compared support use after different modes of sudden bereavement, to test a specific hypothesis about inequities in support for people bereaved by suicide.
- Given the possibility of selection bias (favouring higher social classes) and male non-response bias, the results of this study may be more generalisable to young bereaved women than men, and to the more highly educated.

Key words:

Suicide; bereavement; suicide prevention; support; unmet needs; stigma.

For peer review only

Introduction

Empirical research now supports an association between bereavement by suicide and a range of negative health outcomes, including an increased probability of suicide[1] and of suicide attempt in close contacts[2]. US and UK suicide prevention strategies recommend providing support for people bereaved by suicide[3-6], but the extent of implementation is unknown. The starting point in addressing this task is to provide a description of the nature of support services currently used. The next challenges are the paucity of trial evidence for effective interventions[7], and the tendency of people bereaved by suicide to avoid seeking help[8;9] despite expressing clear unmet needs[10]. This avoidance is likely to be linked to stigmatising societal beliefs about suicide as a failure of problem-solving[9]. High levels of stigma relative to other bereaved groups[11] may reduce both willingness to seek help and friends' or relatives' readiness to offer support[9;12]. This is concerning if stigma adversely affects access to support in a population vulnerable to suicide[13].

No British study has provided an overview of the range of support received by people bereaved by suicide. US surveys have tended to be small and localised[14-16] or involve help-seeking samples[17]. Registry-based studies describe health service use[18-21] but not informal support: a resource known to be valued after suicide bereavement[10]. For service planning purposes we lack population-based studies describing the prevalence and correlates of support received by people bereaved by suicide. Our objective was to address this by conducting a nationwide population-based survey of bereaved adults, collecting data on health outcomes and support received after sudden bereavement. We focused on young adults given concerns about their vulnerabilities to suicide[22] and their tendency to avoid accessing mental health services[23]. We aimed to answer the following research questions about people bereaved by suicide, compared with those bereaved by other sudden forms of death: whether

For peer review only - http://bmjopen.bmj.com/site/about/guidelines.xhtml

they are less likely to receive formal and informal support and more likely to receive no support or delayed support; whether they are more likely to rely exclusively on formal support; whether perceived stigma accounts for reduced receipt of support; and whether there are gender differences in support received.

Method

Patient involvement

Our research question was prompted by UK suicide prevention strategies[4-6] and developed in consultation with a group of bereaved adults and bereavement counsellors. This consultation group identified important outcomes to capture in relation to the impact of sudden bereavement and provision of support, and reviewed successive drafts of the survey questionnaire. This questionnaire was piloted with individuals accessing support from four national bereavement support organisations: Cruse Bereavement Care, Samaritans, Survivors of Bereavement by Suicide, and Widowed by Suicide. Patients were not involved in the population-based recruitment of this study or data analysis. All bereaved individuals participating in the survey were invited to provide contact details for dissemination of study findings, and to bookmark the findings section of the study website: http://www.ucl.ac.uk/psychiatry/bereavementstudy .

Study design and participants

We conducted a national cross-sectional survey of young adults working or studying at UK higher education institutions (HEIs), avoiding the biases associated with recruiting a help-seeking sample[24]. In 2010 all 164 HEIs in the UK were invited to participate, following up non-responding HEIs to encourage broad socioeconomic and geographic representation. Over 20% (37/164) agreed, with a higher response (40%) from those classified as the more

For peer review only - http://bmjopen.bmj.com/site/about/guidelines.xhtml

prestigious Russell Group universities. This accessed an estimated sampling frame of 659,572 staff and students. The majority of participating HEIs followed study protocol in sending an individual email invitation, with embedded survey link, to each staff and student member. For reasons of sensitivity ten HEIs modified this strategy, either by emailing students only, using their weekly news digest email, or advertising via staff and student intranet. All recipients, whether bereaved or not, were invited to take part in a survey of "the impact of sudden bereavement on young adults", with the aim of masking them to the specific study hypotheses. As the denominator of bereaved people could not be ascertained using survey methods or routine data, there was no accurate way of measuring the proportion of bereaved people who responded.

Inclusion criteria were people aged 18-40 who had experienced sudden bereavement of a close friend or relative. Early childhood bereavements (before age 10) were excluded to minimise recall bias. Sudden bereavement was defined as "a death that could not have been predicted at that time and which occurred suddenly or within a matter of days". Exposure status was sub-classified by self-report as: bereavement by suicide, bereavement by sudden unnatural causes (eg. accidental death), and bereavement by sudden natural causes (eg. cardiac arrest). For respondents who had experienced more than one type of sudden bereavement, we categorised exposure as follows: all those bereaved by suicide were classified as such, regardless of other exposures. Those bereaved by non-suicide death were asked to relate their responses to whichever person they had felt closest to, with exposure status classified accordingly. We based our sample size calculation on the primary outcome for a separate study investigating the association between suicide bereavement and suicide attempt[2], indicating that at least 466 participants were required in any one group (two-tailed analysis; 90% power).

For peer review only - http://bmjopen.bmj.com/site/about/guidelines.xhtml

For peer review only

The study was approved by the UCL Research Ethics Committee in 2010 (ref: 1975/002). All participants provided online informed consent.

Procedures

Our online questionnaire[2] elicited quantitative data on socio-demographic and clinical characteristics. We described past suicidal ideation, suicide attempt, and non-suicidal self-harm using standardised measures from the Adult Psychiatric Morbidity Survey (APMS)[25], which distinguishes suicide attempt from non-suicidal self-harm on the basis of intent[26]. We qualified whether each had occurred before or after the bereavement, or both. Depression was measured using the Composite International Diagnostic Interview (CIDI) screen for lifetime depression[27], qualified as above. Perceived stigma, the subjective awareness of others' stigmatising attitudes, was measured using the stigmatisation subscale of the Grief Experience Questionnaire (GEQ)[28]. Likert-style responses to 10 items (e.g. "Since the death how often did you feel avoided by friends?") generated scores of 5-25. We used a fixed-choice question to ascertain the stage at which respondents felt they had been most affected by the loss.

Two tick-box questions probed help received, whether sought or offered, after the bereavement: "How long after the death did you receive help that was valuable to you?"; "What help did you receive after the death? (with ten options, including None and Other – please state). Two tick-box questions probed help-seeking for self-harm: "If you have harmed yourself since the bereavement did you seek help from anyone?"; "Who did you try to get help from?" (with five options, including Other – please state). We derived our seven binary outcomes from responses to these questions.

Our two primary outcomes were: receipt of any formal bereavement support, and receipt of any informal bereavement support. Formal and informal support classifications were derived

For peer review only - http://bmjopen.bmj.com/site/about/guidelines.xhtml

from similar British[23] and international studies of service use[29]. Self-help was considered a separate category due to problematic formal/informal categorisation in relation to bereavement support[30].

Four secondary outcomes were: receipt of no valuable support; immediate receipt (within one week) of valuable support; delayed receipt (beyond six months) of valuable support; and exclusive use of formal support. These thresholds were agreed on the basis of clinical judgement and the published literature[31]. A fifth secondary outcome was whether those who had attempted suicide post-bereavement had sought help for this.

Statistical analysis

We summarised sample characteristics by exposure group using chi-square testing (categorical variables) and one-way analysis of variance (continuous variables). We used multivariable random effects logistic regression to estimate the strength of the associations between mode of bereavement exposure (sudden natural causes/sudden unnatural causes/suicide) and binary outcomes. Our multivariable models included eight pre-specified confounding variables identified from existing literature and clinical judgement: age, gender, socio-economic status, pre-loss depression, pre-loss suicidal and non-suicidal self-harm, other family history of suicide (excluding index bereavement), time since bereavement, and kinship to the deceased. We used HEI as random effect to take account of clustering effects at the institutional level.

For each outcome we conducted two distinct comparisons. The first controlled for the sudden nature of the death, using people bereaved by sudden natural causes as reference category. The second controlled for the violence of the death, using people bereaved by sudden unnatural causes as reference category.

For peer review only - http://bmjopen.bmj.com/site/about/guidelines.xhtml

To test whether stigma attenuated any associations between bereavement exposure and outcomes, we added perceived stigma to our final models.

We added an interaction term to our final models to test a further pre-specified hypothesis: that the effect of bereavement on receipt of support varied by gender such that men bereaved by suicide might show a more marked lack of formal and informal support.

Finally, we conducted sensitivity analyses to assess the impact of simulating predicted non-response biases; excluding 918 respondents from the ten HEIs that had modified the protocol recruitment method.

All analyses were conducted using Stata version 12[32] and complete case analysis.

Results

Of the 659,572 bereaved and non-bereaved people invited to take part, 5,085 people responded to the questionnaire by clicking on the survey link. Of these, 91% (n=4,630) consented to participate, and 68% (n=3,432) fulfilled eligibility criteria (Figure 1). Cluster (HEI) size varied from 3-364 participants (median=52; inter-quartile range=25-120). Missing data for model covariates and outcomes were less than 7% for covariates and less than 4% for outcomes.

The sample was primarily female, white, and blood-related to the deceased (Table 1). There were no statistically significant group differences by bereavement exposure in relation to gender, age, ethnicity, socio-economic status, personality disorder screen[33], or perceived level of social support. The mean time elapsed since bereavement was 4·9 years (SD=5·3; range=1 day to 30 years), with no significant group differences. One quarter (24%; 824/3,432) reported that they had been most affected in the first week after the loss, but a third (38%; 1,274/3,432) endorsed over six months after the loss, with no evidence for group differences.

For peer review only - http://bmjopen.bmj.com/site/about/guidelines.xhtml

Overall 78% (2,572/3,432) of the sample reported receiving some form of support after the loss, whether informal (51%), formal (14%), or both (35%), and 85% (2,173/2572) perceived some aspect of it to have been valuable. Two fifths (42%; 1,438/3,432) had received valuable support within a week of the loss. Overall 20% of the sample received no support at all, excluding the 20 individuals who specified that they had chosen to handle the bereavement alone. The most endorsed source of informal support was family and friends (64%), and of formal support were funeral directors (14%) and health professionals (13%). Self-help was used by 10% (Table 2).

Overall 6% reported having attempted suicide since the bereavement, of which 67% (141/210) had not sought help for any episode of self-harm occurring post-bereavement (Table 2). In those who had sought help, the most common source was a general practitioner (GP) (20%).

People bereaved by suicide were significantly less likely to receive informal support than those bereaved by sudden natural causes (Table 2: adjusted odds ratio (AOR)=0.79; 95% CI=0.64-0.98), and those bereaved by unnatural causes (Table 3: AOR=0.74; 95% CI=0.58-0.96). People bereaved by sudden unnatural causes were significantly more likely to receive formal bereavement support than those bereaved by sudden natural causes (AOR=1.28; 95% CI=1.05-1.56), but there were no other group differences on this outcome.

Compared with people bereaved by sudden natural mortality causes, people bereaved by suicide were significantly less likely to receive immediate support (Table 2: AOR=0.73; 95% CI=0.59-0.90) and significantly more likely to report delayed receipt of support (AOR=1.33; 95% CI=1.08-1.64). There were no other group differences on this or any other secondary outcome.

For peer review only - http://bmjopen.bmj.com/site/about/guidelines.xhtml

After adding perceived stigma to models, all four significant associations of suicide bereavement with support outcomes became non-significant, as did the association between bereavement by sudden unnatural causes and use of formal bereavement support.

Interaction tests showed no evidence that gender modified any of the associations identified.

Significant associations between suicide bereavement and outcomes were unchanged in sensitivity analyses simulating predicted non-response biases. Only the association between bereavement by sudden unnatural causes and use of formal bereavement support became non-significant.

Discussion

Main findings

One in four people bereaved by suicide in this national sample had received no formal or informal support after their loss, despite the major emphasis in English[4], Northern Irish[5] and Welsh[6] suicide prevention strategies on improved suicide bereavement support. Not only were people bereaved by suicide significantly less likely to have received informal support, they were also more likely to describe delays in receiving any formal or informal support. These findings may not reflect preferences, as receipt of support is a function of what is perceived to be available. It is therefore unclear whether our findings reflect reduced help-seeking or an objective lack of help offered. The cross-sectional, observational nature of these data limits causal inference. However, surveys of the perceived needs of people bereaved by suicide indicate clear unmet needs for social networks to respond proactively and empathically, and for professionals to offer immediate outreach[10]. This suggests that our findings represent gaps in support rather than a rejection or avoidance of help. Whether stigma explains the inequalities observed, perhaps by inhibiting help-seeking or offers of

For peer review only - http://bmjopen.bmj.com/site/about/guidelines.xhtml

support, requires further research. The low rates of help-seeking after suicide attempt are particularly concerning in people bereaved by suicide given their higher risk of suicide attempt[2] and the high priority accorded to their needs for support within British suicide prevention strategies.

Results in the context of other studies

Perhaps reflecting cultural differences, our findings differ from those of a representative US sample of suicide-bereaved relatives, in which 24% had received either formal or informal support and 33% preferred to cope without assistance[34]. In a US help-seeking sample 78% reported receiving individual therapy after suicide bereavement[17]; a proportion greatly exceeding formal support use in our population-based sample. The only British study of support after suicide[35] did not state the overall proportion receiving support, but the prevalence of counselling matched that in our study. Consultation with faith leaders was double that in our sample, perhaps reflecting differing age profiles. Studies comparing groups bereaved by suicide and other causes have only focussed on single measures of perceived social support and have, like our study, found weak or no evidence for group differences[14-16].

Strengths and limitations

This national sample represents the largest and most comprehensive study of support received by people bereaved by a close contact's sudden death. It included respondents who were related and unrelated to the deceased, recognising that adverse outcomes and needs for support apply regardless of kinship[2]. In conducting specific group comparisons we were able to ascertain that reduced receipt of informal support was attributable to suicide bereavement rather than unnatural losses more widely. Results were robust to sensitivity analysis, and use of a precise sampling frame allowed us to be clear about the limits of generalisability. The possibility of selection bias through sampling from HEIs, and the pronounced male non-

For peer review only - http://bmjopen.bmj.com/site/about/guidelines.xhtml

response bias limit generalizability beyond highly-educated female groups. Without a denominator we were unable to present a response rate, but assume that the majority of non-responders were non-bereaved. It was possible that those worst affected had biased recall of support received and its value.

Policy implications

The quarter of our suicide-bereaved sample who received no support represent failed implementation of UK suicide prevention strategies[4-6]. This group was distinct from the 1% who stated that they preferred to cope without assistance. The inequities in informal support we identified for people bereaved by suicide suggest a need for psychosocial interventions to address social avoidance and stigmatising attitudes within social networks. Public education to raise awareness of the vulnerabilities of people bereaved by suicide, the range of support available[36], and advice on how to support them[36;37] could encourage social networks to respond more readily after suicide loss. This, along with interventions to address self-stigma, might also encourage the bereaved to seek help by reinforcing the idea that they are worthy of support. Current UK developments in national systems of early outreach after suicide[38] will address the identified delays in support, particularly at a stage when motivation and awareness is low[10].

Further research

Research is needed to explore the influence of stigma on willingness to seek help after suicide bereavement, and on others' readiness to offer support. Thematic analysis of our qualitative survey data will permit a more nuanced understanding of this. Studies that deepen our understanding of help-seeking preferences after suicide attempt in people bereaved by suicide might help address risk of re-attempt. Expanding the limited evidence base for

For peer review only - http://bmjopen.bmj.com/site/about/guidelines.xhtml

interventions after suicide bereavement[7] is important, as is investigating the potential for adverse psychological effects of early[39] and group[40] interventions.

Conclusions

Our study demonstrated clear inequities in the support received by people bereaved by the suicide of a close contact, manifested in delayed receipt of support and a lesser likelihood of receiving support from family and friends. It is concerning that two-thirds of a group featuring so prominently in UK suicide prevention strategies receive no formal support, and that a quarter receive no support at all. Those responsible for implementing suicide prevention strategies should commission lay guidance on how to support someone bereaved by suicide, and improve national systems of immediate outreach after suicide loss.

Acknowledgements:

We would like to thank all the HEIs from England, Wales, Northern Ireland, and Scotland that consented to participate, listed below, and all the bereaved individuals who took time to respond to the online survey.

Participating HEIs: Bishop Grosseteste University College Lincoln; Bournemouth University; Central School of Speech and Drama; City University; Cranfield University; Courtauld Institute; De Montfort University; University of Greenwich; King's College London; Liverpool Institute for Performing Arts; Liverpool John Moores University; London Metropolitan University; Norwich University College of the Arts; Royal Veterinary College; School of Oriental and African Studies; St George's, University of London; Staffordshire University; Trinity Laban Conservatoire of Music and Dance; UCL; University Campus Suffolk; University of Bedfordshire; University of Chester; University of Cumbria; University of Leeds; University of Liverpool; University of Oxford; University of

For peer review only

For peer review only - http://bmjopen.bmj.com/site/about/guidelines.xhtml

Southampton; University of Worcester; University of Westminster; Queen Margaret University; Heriot-Watt University; Scottish Agricultural College; University of Dundee; Cardiff University; Cardiff Metropolitan University (formerly University of Wales Institute Cardiff); Queen's University Belfast; University of Ulster.

Contributors: AP, DO, and MK had the idea for and designed the study. PM and LS contributed to questionnaire design and recruitment strategy. AP recruited participants, managed the survey, and collected and cleaned data. AP, KR, LM and DO conducted (and are responsible for) data analysis. All authors interpreted data, contributed to writing of the report and approved the final version before submission. AP conducted the literature search. AP had full access to all the data in the study and takes responsibility for the integrity of the data and the accuracy of the data analysis. No participant is identifiable from the analysis or study report. AP is the guarantor.

Ethical approval: This project was independently peer reviewed by the Medical Research Council (MRC). Ethical approval was granted by the UCL Research Ethics Committee in 2010 (reference number: 1975/002).

Funding: This project was funded by a MRC Population Health Scientist Fellowship to AP (G0802441). The MRC had no role in design and conduct of the study; collection, management, analysis, and interpretation of the data; or preparation, review, and approval of the manuscript. The views expressed are those of the authors and not necessarily those of the Medical Research Council.

Transparency: The lead author (AP) affirms that the manuscript is an honest, accurate, and transparent account of the study being reported; that no important aspects of the study have been omitted; and that any discrepancies from the study as planned have been explained.

For peer review only - http://bmjopen.bmj.com/site/about/guidelines.xhtml

Data sharing: Informed consent for collecting patient-level data was obtained on the basis that data would be anonymised, stored in accordance with the Data Protection Act 1998, only used for the purposes of the study, and not transferred to an organisation outside UCL. Requests to collaborate on analysis of anonymised data with low risk of identification should be made by contacting the corresponding author.

Competing interests: All authors have completed the ICMJE uniform disclosure form at www.icmje.org/coi_disclosure.pdf and declare: financial support from the Medical Research Council for the submitted work; no financial relationships with any organisations that might have an interest in the submitted work in the previous three years; no other relationships or activities that could appear to have influenced the submitted work.

Copyright: The Corresponding Author has the right to grant on behalf of all authors and does grant on behalf of all authors, an exclusive licence on a worldwide basis to the BMJ Publishing Group Ltd to permit this article (if accepted) to be published in BMJ editions and any other BMJPGL products and sublicences such use and exploit all subsidiary rights, as set out in their licence.

Supplementary files:

Research checklist: STROBE statement

For peer review only

Table 1: Characteristics of participants by type of bereavement exposure

Participants bereaved by:	sudden natural death (n=2106)	sudden unnatural death (n=712)	suicide (n=614)	Total (n=3,432)	p-value [a]
**Socio-demographic characteristics**					
**Gender †**					
Female n (%)	1709 (81)	576 (81)	499 (81)	2784 (81)	0·955
Missing n (%)	1 (<1)	0 (0)	0 (0)	1 (<1)	
**Age of participant †** mean (SD)	24·9 (6·3)	25·2 (6·3)	25·2 (6·0)	25·0 (6·3)	0·069
**Self-defined ethnicity**					
white n (%)	1877 (89)	645 (91)	562 (92)	3084 (90)	0·102
missing n (%)	1 (<1)	2 (<1)	0 (0)	3 (<1)	
**Socio-economic status [b] †**					
social classes 1.1 & 1.2 n (%)	603 (29)	224 (32)	176 (29)	1003 (29)	0·604
social class 2 n (%)	684 (33)	234 (33)	204 (33)	1122 (33)	
social class 3 n (%)	259 (12)	77 (11)	68 (11)	404 (12)	
social class 4 n (%)	90 (4)	34 (5)	32 (5)	156 (5)	
social classes 5,6,7 & 9 n (%)	409 (19)	115 (16)	113 (18)	638 (19)	
missing n (%)	61 (3)	27 (4)	21 (3)	109 (3)	
**Educational status**					
attained up to 2° school leaving qualification n (%)	964 (46)	286 (40)	255 (42)	1505 (44)	**0·035**
attained degree or above n (%)	1136 (54)	424 (60)	359 (59)	1919 (56)	
missing n (%)	6 (<1)	2 (<1)	0 (0)	8 (<1)	

For peer review only - http://bmjopen.bmj.com/site/about/guidelines.xhtml

Participants bereaved by:	sudden natural death (n=2106)	sudden unnatural death (n=712)	suicide (n=614)	Total (n=3,432)	p-value [a]
**Student status**					
student n (%)	1797 (85)	613 (86)	526 (86)	2936 (86)	0·905
staff n (%)	253 (12)	78 (11)	68 (11)	399 (12)	
both n (%)	55 (3)	21 (3)	20 (3)	96 (3)	
missing n (%)	1 (<1)	0 (0)	0 (0)	1 (<1)	
**Measure of social support [c]**					
no lack of perceived social support n (%)	1234 (59)	411 (58)	345 (56)	1990 (58)	0·297
moderate lack of perceived social support n (%)	549 (26)	197 (28)	168 (27)	914 (27)	
severe lack of perceived social support n (%)	323 (15)	102 (14)	100 (16)	525 (15)	
missing n (%)	0 (0)	2 (<1)	1 (<1)	3 (<1)	
**Clinical characteristics**					
**Personality disorder screen positive [d]**					
Yes n (%)	743 (35)	227 (32)	225 (37)	1195 (35)	0·071
missing n (%)	131 (6)	31 (4)	33 (5)	195 (6)	
**Family history of psychiatric problems**					
Yes n (%)	1243 (59)	434 (61)	412 (67)	2089 (61)	**0·005**
missing n (%)	153 (7)	41 (6)	39 (6)	233 (7)	
**Other family history of suicide †**					
Yes n (%)	123 (6)	41 (6)	53 (7)	217 (6)	0·071
missing n (%)	158 (8)	43 (6)	40 (7)	241 (7)	

Participants bereaved by:	sudden natural death (n=2106)	sudden unnatural death (n=712)	suicide (n=614)	Total (n=3,432)	p-value [a]
**Pre-loss non-suicidal self-harm & suicide attempt †**					
Yes n (%)	434 (21)	134 (19)	150 (24)	718 (21)	0·050
missing n (%)	157 (8)	41 (6)	41 (7)	239 (7)	
**Pre-loss depression †**					
Yes n (%)	370 (18)	129 (18)	143 (23)	642 (19)	**0·015**
missing n (%)	85 (4)	21 (3)	24 (4)	130 (4)	
**Characteristics of the bereavement**					
**Kinship to the deceased †**					
blood relative n (%)	1786 (85)	351 (49)	296 (48)	2433 (71)	**<0·001**
unrelated n (%)	313 (15)	356 (50)	317 (52)	980 (29)	
missing n (%)	7 (<1)	5 (1)	1 (<1)	13 (<1)	
**Age of the deceased**: mean (SD)	55.1 (21.5)	31·0 (17·4)	31·9 (15·2)	45·9 (22·8)	**<0·001**
**Time since bereavement †** : mean (SD)	4·8 (5·3)	5·3 (5·4)	5·1 (5·0)	5·0 (5·3)	0·140
**Bereavement in last 2 years**					
Yes n (%)	707 (34)	186 (26)	168 (27)	1061 (31)	**<0·001**
No n (%)	1399 (67)	526 (74)	446 (73)	2371 (69)	
**GEQ stigmatization subscale score** *mean (SD)*	11·9 (3·8)	12·3 (4.0)	14·0 (4·3)	12·3 (4·0)	**<0·001**

For peer review only - http://bmjopen.bmj.com/site/about/guidelines.xhtml

Participants bereaved by:	sudden natural death (n=2106)	sudden unnatural death (n=712)	suicide (n=614)	Total (n=3,432)	p-value [a]
**Time point rated as worst stage after the loss**					
within a week (%)	560 (25)	156 (22)	148 (24)	824 (24)	0.112
up to a month n (%)	330 (16)	92 (13)	81 (13)	503 (15)	
up to 6 months n (%)	330 (16)	122 (17)	112 (18)	564 (16)	
up to a year n (%)	359 (17)	147 (21)	101 (17)	607 (18)	
up to 3 years n (%)	216 (10)	80 (11)	69 (11)	365 (11)	
over 3 years n (%)	181 (9)	62 (9)	59 (10)	302 (9)	
missing n (%)	170 (8)	53 (8)	44 (7)	267 (8)	
**Bereavement support**					
**Any formal/informal support [e] received after bereavement**					
Yes n (%)	1573 (75)	558 (78)	441 (72)	2572 (75)	**0.031**
No n (%)	446 (21)	131 (18)	148 (24)	725 (21)	
Missing n (%)	87 (4)	23 (3)	25 (4)	135 (4)	
**Formal/informal support perceived to be valuable** (of n=2572)					
Yes n (%)	1,335 (85)	464 (83)	374 (85)	2173 (85)	0.621
No n (%)	216 (14)	85 (15)	59 (13)	360 (14)	
Missing n (%)	22 (1)	9 (2)	8 (2)	39 (2)	
**Type of formal/informal support received** (of n=2572)					
Formal only n (%)	217 (14)	76 (14)	68 (15)	361 (14)	0·922
Informal only n (%)	796 (51)	286 (51)	220 (50)	1302 (51)	
Both formal and informal n (%)	560 (36)	196 (35)	153 (35)	909 (35)	

For peer review only - http://bmjopen.bmj.com/site/about/guidelines.xhtml

Participants bereaved by:	sudden natural death (n=2106)	sudden unnatural death (n=712)	suicide (n=614)	Total (n=3,432)	p-value [a]
**Point at which any valuable support received after loss**					
within a day n(%)	623 (30)	234 (33)	150 (24)	1007 (29)	**0·001**
within a week n(%)	290 (14)	72 (10)	69 (11)	431 (13)	
within a month n(%)	154 (7)	50 (7)	44 (7)	248 (7)	
within 6 months n(%)	117 (6)	35 (5)	46 (8)	198 (6)	
within a year n(%)	58 (3)	31 (4)	15 (2)	104 (3)	
over a year n(%)	124 (6)	49 (7)	58 (10)	231 (7)	
at no point n(%)	632 (30)	211 (30)	198 (32)	1041 (30)	
missing n (%)	108 (5)	30 (4)	34 (6)	172 (5)	
**Whether help sought after self-harm post-bereavement [f]**					
Yes n (%)	42 (38)	8 (19)	19 (34)	69 (33)	0.093
No n (%)	70 (63)	34 (81)	37 (66)	141 (67)	

† = pre-specified confounding variable used in adjusted models
[a] significance threshold of p=0·05; not adjusted for multiple testing
[b] socio-economic status using the 5 categories from UK Office for National Statistics
[c] measure of social support from Adult Psychiatric Morbidity Survey (25)
[d] SAPAS-SR screen for personality disorder (33)
[e] excluding self-help
[f] in sub-sample of n=210 who had made a suicide attempt since the index bereavement

For peer review only - http://bmjopen.bmj.com/site/about/guidelines.xhtml

Table 2: Specific type of support used after bereavement

Participants bereaved by:	sudden natural death (n=2106) n (% of exposure group)	sudden unnatural death (n=712) n (% of exposure group)	suicide (n=614) n (% of exposure group)	Total (n=3,432) n (% of total sample)
**Specific bereavement support reported** [a]				
**Formal support**				
health services (doctor, nurse, therapist, counsellor)	283 (13)	86 (12)	83 (14)	452 (13)
social services	0 (0)	0(0)	1(<1)	1 (<1)
private counsellor or therapist	171 (8)	78 (11)	73 (12)	322 (9)
voluntary sector services (helpline, counsellor)	120 (6)	53 (7)	51 (8)	224 (7)
police officers	77 (4)	102 (14)	45 (7)	224 (7)
funeral directors	359 (17)	85 (12)	51 (8)	495 (14)
coroners' officers	130 (6)	51 (7)	35 (6)	216 (6)
school teachers or school counselling services	28 (1)	11 (2)	9 (2)	48 (1)
college tutor or college counselling services	34 (2)	11 (2)	19 (3)	64 (2)
line manager or employee counselling services	5 (<1)	3 (<1)	1 (<1)	9 (<1)
Subtotal formal support	1207 (57)	480 (67)	368 (60)	2055 (60)
**Informal support**				
friends and family	1349 (64)	481 (68)	370 (60)	2200 (64)
spiritual/religious advisors	40 (2)	10 (1)	10 (2)	60 (2)
complementary and alternative medicine (CAM)	1 (<1)	0 (0)	0 (0)	1 (<1)
Subtotal informal support	1390 (66)	491 (69)	380 (62)	2261 (66)

For peer review only - http://bmjopen.bmj.com/site/about/guidelines.xhtml

Participants bereaved by:	sudden natural death (n=2106) n (% of exposure group)	sudden unnatural death (n=712) n (% of exposure group)	suicide (n=614) n (% of exposure group)	Total (n=3,432) n (% of total sample)
**Other**				
self-help (website, book, leaflet)	208 (10)	61 (9)	79 (13)	348 (10)
Specific source not specified	23 (1)	7 (1)	6 (1)	36 (1)
Other (not classified as above)[b]	3 (<1)	2 (<1)	1 (<1)	6 (<1)
Subtotal Other	234 (11)	70 (10)	86 (14)	390 (11)
**None**				
Chose to handle it alone[c]	15 (<1)	4 (1)	1 (<1)	20 (1)
No help received[d] n(%)	428 (20)	129 (18)	141 (23)	698 (20)
**Specific support sought following any self-harm post-bereavement**[e]				
none	70 (63)	34 (81)	37 (66)	141 (67)
friend	18 (16)	2 (5)	8 (14)	28 (13)
family member	13 (12)	3 (7)	7 (13)	23 (11)
general practitioner (GP)	25 (22)	5 (12)	12 (21)	42 (20)
hospital professionals	10 (9)	1 (2)	5 (9)	16 (8)
counsellor	9 (8)	1 (2)	4 (7)	13 (6)
mental health team member	2 (2)	0 (0)	3 (5)	5 (2)
voluntary sector organisation	1 (1)	0 (0)	0 (0)	1 (<1)
school/college teaching staff	2 (2)	0 (0)	0 (0)	1 (<1)

For peer review only - http://bmjopen.bmj.com/site/about/guidelines.xhtml

[a] categories not mutually exclusive
[b] category included organisations such as the diplomatic service, shipping services (for repatriating the body), and employees at the deceased's bank.
[c] 16/20 people in this category also endorsed other sources of formal or informal support
[d] category excluded those who had used self-help and those who indicated they had chosen to handle the bereavement alone
[e] in the n=210 individuals who had attempted suicide post-bereavement; categories not mutually exclusive

For peer review only

For peer review only - http://bmjopen.bmj.com/site/about/guidelines.xhtml

Table 3: Estimates of the relationship between support outcomes and bereavement exposure (suicide *versus* sudden natural death)

**Exposure group**	**Sudden natural death (n = 2106)**		**Sudden unnatural death (n = 712)**					**Suicide (n = 614)**				
**Primary outcomes**	**Prevalence n (%)**	**Odds ratio** (reference)	**Prevalence n (%)**	**Unadjusted odds ratio (95% CI)**	***p* value***	**Adjusted[a] odds ratio (95% CI)**	***p* value***	**Prevalence n (%)**	**Unadjusted odds ratio (95% CI)**	***p* value***	**Adjusted[a] odds ratio (95% CI)**	***p* value***
receipt of formal support post-bereavement	776 (37)	1	272 (38)	1.05 (0.88-1.27)	0.548	**1.28† (1.05-1.56)**	**0.015**	221 (36)	0.97 (0.80-1.18)	0.753	1.17 (0.94-1.44)	0.155
receipt of informal support post-bereavement	1396 (66)	1	491 (69)	1.13 (0.92-1.38)	0.257	1.06 (0.86-1.33)	0.553	389 (63)	0.83 (0.68-1.02)	0.083	**0.79† (0.64-0.98)**	**0.038**
**Secondary outcomes**												
no support post-bereavement[b]	428 (20)	1	129 (18)	0.83 (0.66-1.05)	0.122	0.83 (0.65-1.07)	0.149	141 (23)	1.21 (0.97-1.52)	0.097	1.21 (0.95-1.55)	0.119
immediate receipt of support (<1 week)	913 (43)	1	306 (43)	0.97 (0.81-1.17)	0.747	0.96 (0.79-1.17)	0.660	219 (36)	**0.74 (0.60-0.90)**	**0.002**	**0.73† (0.59-0.90)**	**0.003**
delayed receipt of valuable support (>6 months)	814 (39)	1	291 (41)	1.05 (0.88-1.270	0.575	1.10 (0.90-1.35)	0.359	271 (44)	1.26 (1.04-1.53)	0.020	**1.33† (1.08-1.64)**	**0.008**
use of formal support exclusively (in sub-set of n=2572 receiving support)	217 (14)	1	76 (14)	0.98 (0.72-1.33)	0.888	1.11 (0.80-1.54)	0.516	68 (15)	1.12 (0.82-1.55)	0.454	1.26 (0.90-1.76)	0.183
help sought for post-bereavement self-harm	42 (38)	1	8 (19)	0.37 (0.15-0.95)	0.038	0.43 (0.16-1.13)	0.086	19 (34)	0.82 (0.41-1.65)	0.579	0.98 (0.44-2.17)	0.953

For peer review only - http://bmjopen.bmj.com/site/about/guidelines.xhtml

(in sub-set of n=210 who had attempted suicide post-loss)													

[a] adjusted for age, gender, socio-economic status, pre-loss depression, pre-loss suicidal and non-suicidal self-harm, other family history of suicide (excluding index bereavement), time since bereavement, and kinship to the deceased. For each model, exposure group sizes exceeded the 466 respondents required for adequate power, even when using complete case analysis.

[c] outcome excluded those who solely endorsed that they chose to handle the bereavement alone

* significance threshold of p=0·05 for primary outcomes and p=0·01 for secondary outcomes

† association was no longer significant when stigma added to final adjusted model

For peer review only

For peer review only - http://bmjopen.bmj.com/site/about/guidelines.xhtml

Table 4: Estimates of the relationship between support outcomes and bereavement exposure (suicide *versus* sudden unnatural death)

**Exposure group**	**Sudden unnatural death (n = 712)**	**Suicide (n = 614)**			
**Primary outcomes**	**Odds ratio** (reference)	**Unadjusted odds ratio (95% CI)**	***p* value***	**Adjusted[a] odds ratio (95% CI)**	***p* value***
receipt of formal support post-bereavement	1	0.92 (0.72-1.16)	0.462	0.91 (0.72-1.16)	0.437
receipt of informal support post-bereavement	**1**	**0.74 (0.58-0.95)**	**0.020**	**0.74† (0.58-0.96)**	**0.022**
**Secondary outcomes**					
no support post-bereavement[b]	1	1.46 (0.95-1.52)	0.010	1.46 (1.09-1.95)	0.011
immediate receipt of support (<1 week)	1	0.76 (0.60-96)	0.021	0.76 (0.60-0.97)	0.025
delayed receipt of valuable support (>6 months)	1	1.19 (0.94-1.51)	0.139	1.21 (0.95-1.54)	0.120
use of formal support exclusively (in sub-set of n=2572 receiving support)	1	1.16 (0.79-1.70)	0.463	1.13 (0.77-1.67)	0.542
help sought for post-bereavement self-harm (in sub-set of n=210 who had attempted suicide post-loss)	1	2.18 (0.79-5-98)	0.131	2.28 (0.78-6.68)	0.132

Footnotes as per Table 3

For peer review only - http://bmjopen.bmj.com/site/about/guidelines.xhtml

Reference List

(1) Pitman A, Osborn DPJ, King MB, Erlangsen A. Effects of suicide bereavement on mental health and suicide risk. *Lancet Psychiatry* 2014;1:86-94.

(2) Pitman AL, Osborn DPJ, Rantell K, King MB. Bereavement by suicide as a risk factor for suicide attempt: a cross-sectional national UK-wide study of 3432 young bereaved adults. *BMJ Open* 2016;6.

(3) U.S.Department of Health and Human Services (HHS) Office of the Surgeon General and National Action Alliance for Suicide Prevention. National Strategy for Suicide Prevention: Goals and Objectives for Action. 2012. Washington, DC: HHS.

(4) Department of Health. Preventing suicide in England: A cross-government outcomes strategy to save lives. 17680, 1-57. 2012. published online 10 September 2012.

(5) Department of Health Social Services and Public Safety. Protect Life - A Shared Vision. The Northern Ireland Suicide Prevention Strategy 2012-2014. 2012.

(6) Welsh Government. Talk To Me 2 - Suicide and Self Harm Prevention Strategy for Wales 2015-2020 . 2015.

(7) McDaid C, Trowman R, Golder S, Hawton K, Sowden A. Interventions for people bereaved through suicide: systematic review. *Br J Psychiatry* 2008;193:438-443.

(8) Diekstra RFW. Suicide and the attempted suicide: An international perspective. *Acta Psychiatr Scand* 1989;80:1-24.

(9) Cvinar JG. Do Suicide Survivors Suffer Social Stigma: A Review of the Literature. *Perspect Psychiatr Care* 2005;41:14-21.

For peer review only - http://bmjopen.bmj.com/site/about/guidelines.xhtml

(10) Dyregrov K. What Do We Know About Needs for Help After Suicide in Different Parts of the World? *Crisis* 2011;32:310-318.

(11) Pitman AL, Osborn DPJ, Rantell K, King MB. The stigma perceived by people bereaved by suicide and other sudden deaths: A cross-sectional UK study of 3432 bereaved adults. *J Psychosom Res* 2016;87:22-29.

(12) Chapple A, Ziebland S, Hawton K. Taboo and the different death? Perceptions of those bereaved by suicide or other traumatic death. *Sociol Health Illn* 2015;37:610-625.

(13) Rusch N, Zlati A, Black G, Thornicroft G. Does the stigma of mental illness contribute to suicidality? *Br J Psychiatry* 2014;205:257-259.

(14) Farberow NL, Gallagher-Thompson D, Gilewski M, Thompson L. The role of social supports in the bereavement process of surviving spouses of suicide and natural deaths. *Suicide Life Threat Behav* 1992;22:107-124.

(15) Seguin M, Lesage A, Kiely MC. Parental bereavement after suicide and accident: a comparative study. *Suicide Life Threat Behav* 1995;25:489-492.

(16) Range L.M., Niss N.M. Long-term bereavement from suicide, homicide, accidents and natural deaths. *Death Stud* 1990;14:423-433.

(17) McMenamy J.M., Jordan JR, Mitchell AM. What do Suicide Survivors Tell Us They Need? Results of a Pilot Study. *Suicide Life Threat Behav* 2008;38:375-389.

(18) Bolton JM, Au W, Leslie WD et al. Parents bereaved by offspring suicide: a population-based longitudinal case-control study. *JAMA Psychiatry* 2013;70:158-167.

For peer review only

(19) Kuramoto S, Runeson B, Stuart EA, Lichtenstein P, Wilcox HC. Time to hospitalization for suicide attempt by the timing of parental suicide during offspring early development. *JAMA Psychiatry* 2013;70:149-157.

(20) Kuramoto SJ, Stuart EA, Runeson B, Lichtenstein P, Langstrom N, Wilcox HC. Maternal or Paternal Suicide and Offspring's Psychiatric and Suicide-Attempt Hospitalization Risk. *Pediatrics* 2010;126:e1026-e1032.

(21) Wilcox H.C., Kuramoto S.J., Lichtenstein P, Langstrom N, Brent D.A., Runeson B. Psychiatric Morbidity, Violent Crime, and Suicide Among Children and Adolescents Exposed to Parental Death. *J Am Acad Child Adolesc Psychiatry* 2010;49:514-523.

(22) Pitman A, Krysinska K, Osborn D, King M. Suicide in young men. *Lancet* 2012;379:2383-2392.

(23) Brown J, Evans-Lacko S, Aschan L, Henderson M, Hatch S, Hotopf M. Seeking informal and formal help for mental health problems in the community: a secondary analysis from a psychiatric morbidity survey in South London. *BMC Psychiatry* 2014;14:1-15.

(24) Pitman AL, Osborn DPJ, King MB. The use of internet-mediated cross-sectional studies in mental health research. *BJPsych Advances* 2015;21:175-184.

(25) McManus S, Meltzer H, Brugha T, Bebbington P, Jenkins R. Adult psychiatric morbidity in England, 2007. Results of a household survey. 1-141. 2009. Leeds, The Health and Social Care Information Centre; National Centre for Social Research; A survey carried out for The NHS Information Centre for health and social care by the National Centre for Social Research.

For peer review only - http://bmjopen.bmj.com/site/about/guidelines.xhtml

(26) Bebbington PE, Minot S, Cooper C et al. Suicidal ideation, self-harm and attempted suicide: Results from the British psychiatric morbidity survey 2000. *European Psychiatry* 2010;25:427-431.

(27) Robins LN, Wing JK, Wittchen H-U et al. The Composite International Diagnostic Interview. An epidemiologic instrument for use in conjunction with different diagnostic systems and in different cultures. *Arch Gen Psychiatry* 1988;45:1069-1077.

(28) Bailley SE, Dunham K, Kral MJ. Factor structure of The Grief Experience Questionnaire (GEQ). *Death Stud* 2000;24:721-738.

(29) Bruffaerts R, Demyttenaere K, Hwang I et al. Treatment of suicidal people around the world. *Br J Psychiatry* 2011;199:64-70.

(30) Stevenson FA, Britten N, Barry CA, Bradley CP, Barber N. Self-treatment and its discussion in medical consultations: how is medical pluralism managed in practice? *Soc Sci Med* 2003;57:513-527.

(31) Stroebe M, Schut H, Stroebe W. Health outcomes of bereavement. *Lancet* 2007;370:1960-1973.

(32) *Stata Statistical Software: Release 12* College Station, TX: StataCorp LP: StataCorp LP; 2011.

(33) Germans S, Van Heck GL, Moran P, Hodiamont PPG. The Self-report Standardized Assessment of Personality-abbreviated Scale: Preliminary results of a brief screening test for personality disorders. *Personality* 2008;2:70-76.

For peer review only

For peer review only - http://bmjopen.bmj.com/site/about/guidelines.xhtml

(34) Provini C, Everett JR, Pfeffer C.R. Adults mourning suicide: self-reported concerns about bereavement, needs for assistance, and help-seeking behavior. *Death Stud* 2000;24:1-19.

(35) Harwood D., Hawton K., Hope T., Jacoby R. The grief experiences and needs of bereaved relatives and friends of older people dying through suicide: a descriptive and case-control study. *J Affect Disord* 2002;72:185-194.

(36) Public Health England, National Suicide Prevention Alliance. Help is at Hand: Support after someone may have died by suicide. 2015. London, HMSO.

(37) Carson J Spencer Foundation, National Action Alliance for Suicide Prevention, American Association of Suicidology. A Manager's Guide to Suicide Postvention in the Workplace: 10 Action Steps for Dealing with the Aftermath of Suicide. 2013. Denver, CO., Carson J Spencer Foundation.

(38) Department of Health. Preventing suicide in England: Two years on. Second annual report on the cross-government outcomes strategy to save lives. SCLGCP/ MHED / 11160. 2015.

(39) Wessely S, Deahl M. Psychological debriefing is a waste of time. *Br J Psychiatry* 2003;183:12-14.

(40) de Groot M, Kollen BJ. Course of bereavement over 8-10 years in first degree relatives and spouses of people who committed suicide: longitudinal community based cohort study. *BMJ* 2013;347.

For peer review only

For peer review only - http://bmjopen.bmj.com/site/about/guidelines.xhtml

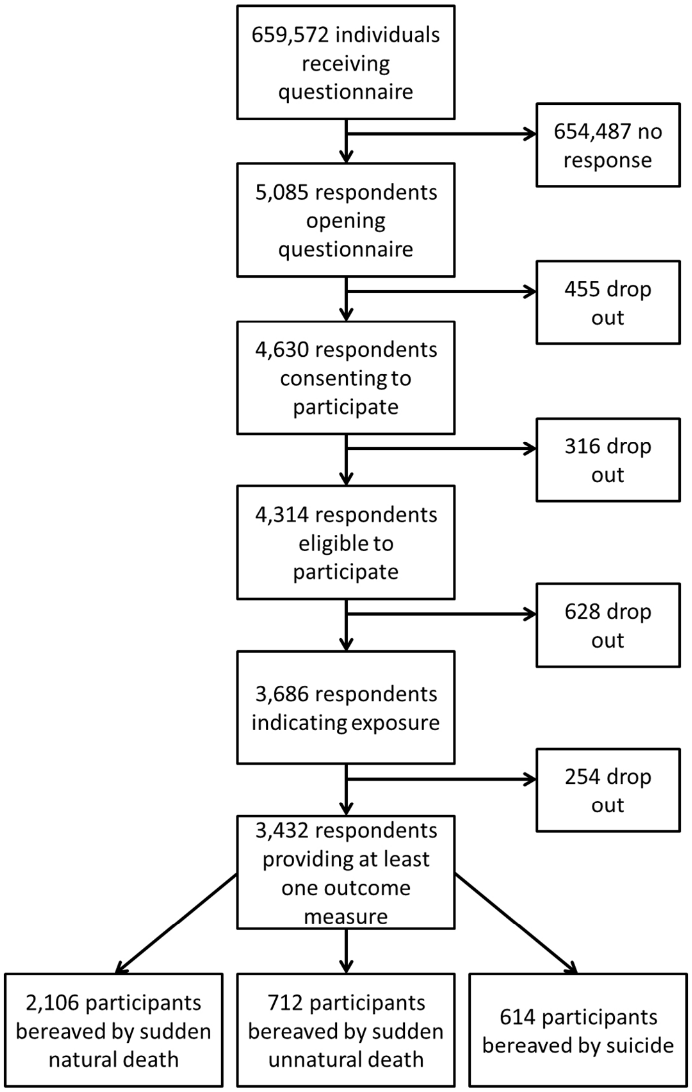

111x159mm (300 x 300 DPI)

STROBE checklist for cross-sectional study of support received after bereavement by suicide and other sudden deaths (UCL Bereavement Study)

Corresponding author: Dr Alexandra Pitman 27 September 2016

Checklist of items that should be included in reports of ***cross-sectional studies***: green denotes page number
http://www.strobe-statement.org/index.php?id=available-checklists

	Item No	Recommendation
**Title and abstract**	1	(*a*) **Indicate the study's design with a commonly used term in the title or the abstract:** abstract/title indicates that we conducted a national cross-sectional study
**Page 1 & 2-3**		(*b*) **Provide in the abstract an informative and balanced summary of what was done and what was found:** abstract outlines our hypothesis, exposures and outcomes, and adjusted odds ratio for the associations hypothesised
**Introduction**		
Background/rationale<br>**Page 4**	2	**Explain the scientific background and rationale for the investigation being reported:** Our introduction outlines the policy context, including key research references, and highlights the lack of evidence to support current suicide prevention strategy.
Objectives<br>**Page 3 & 4**	3	**State specific objectives, including any prespecified hypotheses:** Objectives and primary hypothesis stated in the Abstract and Introduction. Our objective was to conduct a population-based survey comparing the support received by people bereaved by different modes of sudden death and to test specific hypotheses regarding inequalities in support received by people bereaved by suicide.
**Methods**		
Study design<br>**Page 5-6**	4	**Present key elements of study design early in the paper:** Cross-sectional survey stated in Methods.
Setting<br>**Page 5-6**	5	**Describe the setting, locations, and relevant dates, including periods of recruitment, exposure, follow-up, and data collection**: Describes emailing individuals at 37 HEIs in 2010 for cross-sectional data collection. Acknowledgement section details the locations of diverse participating HEIs.
Participants<br>**Page 5-6**	6	**Give the eligibility criteria, and the sources and methods of selection of participants:** Eligibility criteria described as: people aged 18-40 who had experienced sudden bereavement of a close friend or relative after ten years of age.
Variables<br>**Page 7-8**	7	**Clearly define all outcomes, exposures, predictors, potential confounders, and effect modifiers. Give diagnostic criteria, if applicable.** All outcomes described, denoting how these were derived. Exposure clearly defined. Eight pre-specified confounding variables defined and justified. Kinship defined as a potential effect modifier.
Data sources/ measurement<br>**Page 5 &7-8**	8*	**For each variable of interest, give sources of data and details of methods of assessment (measurement). Describe comparability of assessment methods if there is more than one group:** Questionnaire development and content described. Same instrument used for all exposure groups.
Bias<br>**Page 5-6**	9	**Describe any efforts to address potential sources of bias:** We describe how we followed-up non-responding HEIs to ensure a diverse representation of HEIs, and how we masked participants to the study hypothesis. We also describe a decision to use two-tailed analysis to reduce inductive bias.
Study size	10	**Explain how the study size was arrived at:** We based our sample size calculation on the primary outcome of a separate study to describe the association between suicide

For peer review only - http://bmjopen.bmj.com/site/about/guidelines.xhtml

**Page 6**		bereavement and suicide attempt (the rarest outcome), to detect a doubling of the UK community prevalence of lifetime suicide attempt (6·5%) in young adult samples.
Quantitative variables<br><br>**Page 5-8**	11	**Explain how quantitative variables were handled in the analyses. If applicable, describe which groupings were chosen and why:** Our Methods section defines the 3 exposure groups, 7 outcomes, and 8 covariates; and how each was used in the analysis.
Statistical methods<br><br>**Page 8-9**	12	**(*a*) Describe all statistical methods, including those used to control for confounding:** We describe our use of multivariable logistic regression, including justification of the 8 covariates used in the adjusted models.
		**(*b*) Describe any methods used to examine subgroups and interactions:** We describe how we tested for an interaction with kinship.
		**(*c*) Explain how missing data were addressed:** We explain that levels of missing data were low (<7% for model covariates, and <4% for outcomes) and so our statistician co-authors advised that we did not need to use best and worst case scenarios to impute missing values as part of our sensitivity analyses.
		**(*d*) If applicable, describe analytical methods taking account of sampling strategy:** We describe our use of a cluster variable to take into account the potential for clustering of responses at the HEI level.
		**(*e*) Describe any sensitivity analyses:** We describe sensitivity analyses that assessed the impact of simulating more stringent inclusion criteria for the sampling strategy.
**Results**		
Participants<br><br>**Page 9, Figure 1**	13*	**(a) Report numbers of individuals at each stage of study—eg numbers potentially eligible, examined for eligibility, confirmed eligible, included in the study, completing follow-up, and analysed:** We specify numbers of those participating, consenting, and eligible, and present the participant flow in Figure 1.
		**(b) Give reasons for non-participation at each stage:** numbers not consenting, not eligible, not indicating exposure group, and not providing at least 1 outcome measure presented in Figure 1.
		**(c) Consider use of a flow diagram**: see Figure 1
Descriptive data<br><br>**Page 9-10, Tables 1 & 2**	14*	**(a) Give characteristics of study participants (eg demographic, clinical, social) and information on exposures and potential confounders:** Tables 1 & 2 and text indicates descriptive characteristics by exposure group.
		**(b) Indicate number of participants with missing data for each variable of interest**: Tables 1 & 2 provide proportion of missing values for each covariate of interest by exposure group.
Outcome data<br>**Table 3**	15*	**Report numbers of outcome events or summary measures:** Table 3 presents prevalence for each outcome by exposure group.
Main results<br><br>**Page 10-11, Tables 3 & 4**	16	**(*a*) Give unadjusted estimates and, if applicable, confounder-adjusted estimates and their precision (eg, 95% confidence interval). Make clear which confounders were adjusted for and why they were included:** Text and Tables 3 and 4 provide unadjusted and adjusted estimates, with 95% confidence intervals and p-values.
		**(*b*) Report category boundaries when continuous variables were categorized**: N/A
		**(*c*) If relevant, consider translating estimates of relative risk into absolute risk for a meaningful time period:** N/A
Other analyses<br><br>**Page 11**	17	**Report other analyses done—eg analyses of subgroups and interactions, and sensitivity analyses:** We report: results of adding stigma to final models, tests for an interaction with gender, sensitivity analyses.
**Discussion**		
Key results<br><br>**Page 11-12**	18	**Summarise key results with reference to study objectives:** The start of our discussion summarises the principle findings in relation to our main hypothesis.
Limitations	19	**Discuss limitations of the study, taking into account sources of potential bias or**

For peer review only - http://bmjopen.bmj.com/site/about/guidelines.xhtml

Page 12-13		**imprecision. Discuss both direction and magnitude of any potential bias:** Our discussion summarises both the strengths and weaknesses of this study, both in comparison with other potential approaches, and other previously-used approaches. We consider the possibility of either over- or under-estimation of risks given specific potential biases.
Interpretation<br>**Page 11-13**	20	**Give a cautious overall interpretation of results considering objectives, limitations, multiplicity of analyses, results from similar studies, and other relevant evidence:** Our discussion compares our findings to the existing literature and comments on the degree to which our findings are consistent with this, and the extent to which they contribute to policy developments in relation to provision of support after suicide bereavement.
Generalisability<br>**Page 12-13**	21	**Discuss the generalisability (external validity) of the study results:** We explore the degree to which findings from a primarily female and highly-educated UK HEI population are generalizable, either in the UK or internationally.
**Other information**		
Funding<br>**Page 15**	22	**Give the source of funding and the role of the funders for the present study and, if applicable, for the original study on which the present article is based:** Our footnotes identify the MRC as the funder, and the limits of their role in this study.

*Give information separately for exposed and unexposed groups.

Note: An Explanation and Elaboration article discusses each checklist item and gives methodological background and published examples of transparent reporting. The STROBE checklist is best used in conjunction with this article (freely available on the Web sites of PLoS Medicine at http://www.plosmedicine.org/, Annals of Internal Medicine at http://www.annals.org/, and Epidemiology at http://www.epidem.com/). Information on the STROBE Initiative is available at www.strobe-statement.org.

For peer review only

BMJ Open

Support received after bereavement by suicide and other sudden deaths: a cross-sectional UK study of 3,432 young bereaved adults

For peer review only

Journal:	*BMJ Open*
Manuscript ID	bmjopen-2016-014487.R1
Article Type:	Research
Date Submitted by the Author:	06-Feb-2017
Complete List of Authors:	Pitman, Alexandra; UCL, UCL Division of Psychiatry<br>Rantell, Khadija; University College London, Institute of Neurology<br>Moran, Paul; University of Bristol<br>Sireling, Lester; independent medico-legal practice<br>Marston, Louise; UCL, Primary Care and Population Health<br>King, Michael; University College Medical School, UCL Division of Psychiatry<br>Osborn, David; UCL, UCL Division of Psychiatry
**Primary Subject Heading**:	Mental health
Secondary Subject Heading:	Epidemiology, Health services research, Mental health, Public health
Keywords:	MENTAL HEALTH, Suicide & self-harm < PSYCHIATRY, PUBLIC HEALTH, Organisation of health services < HEALTH SERVICES ADMINISTRATION & MANAGEMENT

SCHOLARONE™
Manuscripts

Support received after bereavement by suicide and other sudden deaths: a cross-sectional UK study of 3,432 young bereaved adults

Dr Alexandra L. Pitman, Honorary Research Associate, UCL Division of Psychiatry, University College London, Maple House, 149 Tottenham Court Rd, London W1T 7NF, UK (corresponding author) a.pitman@ucl.ac.uk

Dr Khadija Rantell, Research Associate, UCL Institute of Neurology, University College London, Queen Square, London WC1N 3BG, UK

Dr Paul Moran, Reader in Psychiatry, School of Social and Community Medicine, University of Bristol, Oakfield House, Oakfield Grove, Clifton, Bristol BS8 2BN, UK

Dr Lester Sireling, Consultant psychiatrist in independent medico-legal practice, c/o UCL Division of Psychiatry, University College London, Maple House, 149 Tottenham Court Rd, London W1T 7NF, UK

Dr Louise Marston, Senior Research Associate, UCL Research Department of Primary Care & Population Health, University College London, Rowland Hill Street, London NW3 2PF, UK

Professor Michael B. King, Professor of Primary Care Psychiatry, UCL Division of Psychiatry, University College London, Maple House, 149 Tottenham Court Rd, London W1T 7NF, UK

Professor David P.J. Osborn, Professor of Psychiatric Epidemiology, UCL Division of Psychiatry, University College London, Maple House, 149 Tottenham Court Rd, London W1T 7NF, UK

For peer review only

For peer review only - http://bmjopen.bmj.com/site/about/guidelines.xhtml

Abstract

Objective: To test the hypothesis that people bereaved by suicide are less likely to receive formal or informal support than people bereaved by other causes of sudden death.

Design: National cross-sectional study.

Setting: Adults working or studying at any UK Higher Education Institution (HEI) in 2010.

Participants: 3,432 eligible respondents aged 18-40 bereaved by the sudden death of a close friend or relative, sampled from approximately 659,572 bereaved and non-bereaved staff and students at 37 of 164 UK HEIs invited to participate.

Exposures: Bereavement by suicide (n=614; 18%), by sudden unnatural causes (n=712; 21%) and by sudden natural causes (n=2106; 61%).

Main outcome measures: Receipt of formal and of informal support post-bereavement; timing of valued support.

Results: 21% (725/3432) of our sample of bereaved adults reported receiving no formal or informal bereavement support, with no evidence for group differences. People bereaved by suicide were less likely to have received informal support than those bereaved by sudden natural causes (adjusted odds ratio (AOR) = 0.79; 95% CI=0.64 to 0.98), or unnatural causes (AOR=0.74; 95% CI=0.58 to 0.96), but did not differ from either comparison group on receipt of formal support. People bereaved by suicide were less likely to have received immediate support (AOR=0.73; 95% CI=0.59 to 0.90) and more likely to report delayed receipt of support (AOR=1.33; 95% CI=1.08 to 1.64) than people bereaved by sudden natural causes. Associations were not modified by gender, or age bereaved, but became non-significant when adjusting for stigma.

For peer review only - http://bmjopen.bmj.com/site/about/guidelines.xhtml

Conclusions: People bereaved by suicide are less likely to receive informal support than people bereaved by other causes of sudden death, and are more likely to perceive delays in accessing any support. This is concerning given their higher risk of suicide attempt and the recommendations within suicide prevention strategies regarding their need for support.

Study registration: http://www.ucl.ac.uk/psychiatry/bereavementstudy/

Strengths and limitations of this study

- We conducted a large population-based closed survey to identify bereaved friends and relatives, avoiding the biases inherent to using a help-seeking sample.
- We captured use of a wide range of formal and informal support sources, and the time taken to access valued support.
- We compared support use after different modes of sudden bereavement, to test a specific hypothesis about inequities in support for people bereaved by suicide.
- Given the age-range sampled, and the possibility of selection bias (favouring higher social classes) and male non-response bias, the results of this study may only be generalisable to young bereaved women and the more highly educated.

Key words:

Suicide; bereavement; suicide prevention; support; unmet needs; stigma.

For peer review only

For peer review only - http://bmjopen.bmj.com/site/about/guidelines.xhtml

Introduction

Empirical research now supports an association between bereavement by suicide and a range of negative health outcomes, including an increased probability of suicide[1] and of suicide attempt in close contacts[2]. US and UK suicide prevention strategies recommend providing support for people bereaved by suicide[3-6], but the extent of implementation is unknown. The starting point in addressing this task is to provide a description of the nature of support services currently used. The next challenges are the paucity of trial evidence for effective interventions[7], and the tendency of people bereaved by suicide to avoid seeking help[8;9] despite expressing clear unmet needs[10]. This avoidance is likely to be linked to stigmatising societal beliefs about suicide as a failure of problem-solving[9]. High levels of stigma relative to other bereaved groups[11] may reduce both willingness to seek help and friends' or relatives' readiness to offer support[9;12]. This is concerning if stigma adversely affects access to support in a population vulnerable to suicide[13].

No British study has provided an overview of the range of support received by people bereaved by suicide. US surveys have tended to be small and localised[14-16] or involve help-seeking samples[17]. Registry-based studies describe health service use[18-21] but not informal support: a resource known to be valued after suicide bereavement[10]. For service planning purposes we lack population-based studies describing the prevalence and correlates of support received by people bereaved by suicide. Our objective was to address this by conducting a nationwide population-based survey of bereaved adults, collecting data on health outcomes and support received after sudden bereavement. We focused on young adults given concerns about their vulnerabilities to suicide[22], their tendency to avoid accessing mental health services[23], and their priority status within UK suicide prevention strategies[4-6]. Surveying this age range also minimised the potential for memory decay, and narrowed

For peer review only - http://bmjopen.bmj.com/site/about/guidelines.xhtml

period effects. We aimed to answer the following research questions about people bereaved by suicide, compared with those bereaved by other sudden forms of death: whether they are less likely to receive formal and informal support and more likely to receive no support or delayed support; whether they are more likely to rely exclusively on formal support; whether perceived stigma accounts for reduced receipt of support; and whether there are gender differences in support received.

Method

Patient involvement

Our research question was prompted by UK suicide prevention strategies[4-6] and developed in consultation with a group of bereaved adults and bereavement counsellors. This consultation group identified important outcomes to capture in relation to the impact of sudden bereavement and provision of support, and reviewed successive drafts of the survey questionnaire. This questionnaire was piloted with individuals accessing support from four national bereavement support organisations: Cruse Bereavement Care, Samaritans, Survivors of Bereavement by Suicide, and Widowed by Suicide. Patients were not involved in the population-based recruitment of this study or data analysis. All bereaved individuals participating in the survey were invited to provide contact details for dissemination of study findings, and to bookmark the findings section of the study website: http://www.ucl.ac.uk/psychiatry/bereavementstudy .

Study design and participants

We conducted a national cross-sectional survey of young adults working or studying at UK higher education institutions (HEIs), avoiding the biases associated with recruiting a help-seeking sample[24]. In 2010 all 164 HEIs in the UK were invited to participate, following up

For peer review only - http://bmjopen.bmj.com/site/about/guidelines.xhtml

non-responding HEIs to encourage broad socioeconomic and geographic representation. Over 20% (37/164) agreed, with a higher response (40%) from those classified as the more prestigious Russell Group universities. This accessed an estimated sampling frame of 659,572 staff and students. The majority of participating HEIs followed study protocol in sending an individual email invitation, with embedded survey link, to each staff and student member. For reasons of sensitivity ten HEIs modified this strategy, either by emailing students only, using their weekly news digest email, or advertising via staff and student intranet. All recipients, whether bereaved or not, were invited to take part in a survey of "the impact of sudden bereavement on young adults", with the aim of masking them to the specific study hypotheses. As the denominator of bereaved people could not be ascertained using survey methods or routine data, there was no accurate way of measuring the proportion of bereaved people who responded.

Inclusion criteria were people aged 18-40 who had experienced sudden bereavement of a close friend or relative. Early childhood bereavements (before age 10) were excluded to minimise recall bias. Sudden bereavement was defined as "a death that could not have been predicted at that time and which occurred suddenly or within a matter of days". Exposure status was sub-classified by self-report as: bereavement by suicide, bereavement by sudden unnatural causes (eg. accidental death), and bereavement by sudden natural causes (eg. cardiac arrest). For respondents who had experienced more than one type of sudden bereavement, we categorised exposure as follows: all those bereaved by suicide were classified as such, regardless of other exposures. Those bereaved by non-suicide death were asked to relate their responses to whichever person they had felt closest to, with exposure status classified accordingly. We based our sample size calculation on the primary outcome for a separate study investigating the association between suicide bereavement and suicide

For peer review only - http://bmjopen.bmj.com/site/about/guidelines.xhtml

For peer review only

attempt[2], indicating that at least 466 participants were required in any one group (two-tailed analysis; 90% power).

The study was approved by the UCL Research Ethics Committee in 2010 (ref: 1975/002). All participants provided online informed consent.

Procedures

Our online questionnaire[2] elicited quantitative data on socio-demographic and clinical characteristics. We described past suicidal ideation, suicide attempt, and non-suicidal self-harm using standardised measures from the Adult Psychiatric Morbidity Survey (APMS)[25], which distinguishes suicide attempt from non-suicidal self-harm on the basis of intent[26]. We qualified whether each had occurred before or after the bereavement, or both. Depression was measured using the Composite International Diagnostic Interview (CIDI) screen for lifetime depression[27], qualified as above. Perceived stigma, the subjective awareness of others' stigmatising attitudes, was measured using the stigma subscale of the Grief Experience Questionnaire (GEQ)[28]. Likert-style responses to 10 items (e.g. "Since the death how often did you feel avoided by friends?") generated scores of 5-25. We used a fixed-choice question to ascertain the stage at which respondents felt they had been most affected by the loss.

Two tick-box questions probed help received, whether sought or offered, after the bereavement: "How long after the death did you receive help that was valuable to you?"; "What help did you receive after the death? (with ten options, including None and Other – please state). Two tick-box questions probed help-seeking for self-harm: "If you have harmed yourself since the bereavement did you seek help from anyone?"; "Who did you try to get help from?" (with five options, including Other – please state). We derived our seven binary outcomes from responses to these questions.

For peer review only - http://bmjopen.bmj.com/site/about/guidelines.xhtml

Our two primary outcomes were: receipt of any formal bereavement support, and receipt of any informal bereavement support. Formal and informal support classifications were derived from similar British[23] and international studies of service use[29]. Self-help was considered a separate category due to problematic formal/informal categorisation in relation to bereavement support[30].

Four secondary outcomes were: receipt of no valuable support; immediate receipt (within one week) of valuable support; delayed receipt (beyond six months) of valuable support; and exclusive use of formal support. These thresholds were agreed on the basis of clinical judgement and the published literature[31]. A fifth secondary outcome was whether those who had attempted suicide post-bereavement had sought help for this.

Statistical analysis

We summarised sample characteristics by exposure group using chi-square testing (categorical variables) and one-way analysis of variance (continuous variables). We used multivariable random effects logistic regression to estimate the strength of the associations between mode of bereavement exposure (sudden natural causes/sudden unnatural causes/suicide) and binary outcomes. Our multivariable models included eight pre-specified confounding variables identified from existing literature and clinical judgement: age, gender, socio-economic status, pre-loss depression, pre-loss suicidal and non-suicidal self-harm, other family history of suicide (excluding index suicide bereavement), time since bereavement, and kinship to the deceased. We used HEI as random effect to take account of clustering effects at the institutional level.

For each outcome we conducted two distinct comparisons. The first controlled for the sudden nature of the death, using people bereaved by sudden natural causes as reference category.

For peer review only - http://bmjopen.bmj.com/site/about/guidelines.xhtml

The second controlled for the violence of the death, using people bereaved by sudden unnatural causes as reference category.

To test whether stigma attenuated any associations between bereavement exposure and outcomes, we added perceived stigma[28] to our final models.

We added an interaction term to our final models to test a further pre-specified hypothesis: that the effect of bereavement on receipt of support varied by gender such that men bereaved by suicide would show a more marked lack of formal and informal support. In a *post hoc* test for interaction, we assessed whether age at bereavement (before or after age 18) influenced receipt of support, such that bereaved children would be better supported.

Finally, we conducted *a priori* sensitivity analyses to assess the impact of simulating predicted non-response biases; excluding 918 respondents from the ten HEIs that had modified the protocol recruitment method. We conducted an additional sensitivity analysis to compare people bereaved by suicide to a reference category of all those bereaved by non-suicide sudden death.

All analyses were conducted using Stata version 12[32] and complete case analysis.

Results

Of the 659,572 bereaved and non-bereaved people invited to take part, 5,085 people responded to the questionnaire by clicking on the survey link. Of these, 91% (n=4,630) consented to participate, and 68% (n=3,432) fulfilled eligibility criteria (Figure 1). Cluster (HEI) size varied from 3-364 participants (median=52; inter-quartile range=25-120). Missing data for model covariates and outcomes were less than 7% for covariates and less than 4% for outcomes.

For peer review only - http://bmjopen.bmj.com/site/about/guidelines.xhtml

The sample was primarily female, white, and blood-related to the deceased (Table 1). There were no statistically significant group differences by bereavement exposure in relation to gender, age, ethnicity, socio-economic status, personality disorder screen[33], or perceived level of social support. The mean time elapsed since bereavement was 4·9 years (SD=5·3; range=1 day to 30 years), with no significant group differences. One quarter (24%; 824/3,432) reported that they had been most affected in the first week after the loss, but a third (38%; 1,274/3,432) endorsed over six months after the loss, with no evidence for group differences.

Overall 78% (2,572/3,432) of the sample reported receiving some form of support after the loss, whether informal (51%), formal (14%), or both (35%), and 85% (2,173/2572) perceived some aspect of it to have been valuable. Two fifths (42%; 1,438/3,432) had received valuable support within a week of the loss. Overall 20% of the sample received no support at all, excluding the 20 individuals who specified that they had chosen to handle the bereavement alone. The most endorsed source of informal support was family and friends (64%), and of formal support were funeral directors (14%) and health professionals (13%). Self-help was used by 10% (Table 2).

Overall 6% reported having attempted suicide since the bereavement, of which 67% (141/210) had not sought help for any episode of self-harm occurring post-bereavement (Table 2). In those who had sought help, the most common source was a general practitioner (GP) (20%).

People bereaved by suicide were significantly less likely to receive informal support than those bereaved by sudden natural causes (Table 3: adjusted odds ratio (AOR)=0.79; 95% CI=0.64-0.98), and those bereaved by unnatural causes (Table 4: AOR=0.74; 95% CI=0.58-0.96). People bereaved by sudden unnatural causes were significantly more likely to receive

For peer review only - http://bmjopen.bmj.com/site/about/guidelines.xhtml

formal bereavement support than those bereaved by sudden natural causes (Table 3: AOR=1.28; 95% CI=1.05-1.56), but there were no other group differences on this outcome.

Compared with people bereaved by sudden natural mortality causes, people bereaved by suicide were significantly less likely to receive immediate support (Table 3: AOR=0.73; 95% CI=0.59-0.90) and significantly more likely to report delayed receipt of support (AOR=1.33; 95% CI=1.08-1.64). There were no other group differences on this or any other secondary outcome. After adding perceived stigma to models, all four significant associations of suicide bereavement with support outcomes became non-significant, as did the association between bereavement by sudden unnatural causes and use of formal bereavement support.

Interaction tests showed no evidence that gender, or childhood *versus* adult bereavement, modified any of the associations identified.

In sensitivity analyses simulating predicted non-response biases the magnitude and direction of significant associations between suicide bereavement and outcomes were unchanged, apart from the association between bereavement by sudden unnatural causes and use of formal bereavement support, which became non-significant. In an analysis comparing suicide bereavement to all non-suicide sudden bereavements we found similar associations, in terms of magnitude and direction, apart from the association of suicide bereavement with one secondary outcome (delayed receipt of valuable support), which became non-significant.

Discussion

Main findings

One in four people bereaved by suicide in this national sample had received no formal or informal support after their loss, despite the major emphasis in English[4], Northern Irish[5] and Welsh[6] suicide prevention strategies on improved suicide bereavement support. Not only

For peer review only - http://bmjopen.bmj.com/site/about/guidelines.xhtml

were people bereaved by suicide significantly less likely to have received informal support, they were also more likely to describe delays in receiving any formal or informal support. These findings may not reflect preferences, as receipt of support is a function of what is perceived to be available. It is therefore unclear whether our findings reflect reduced help-seeking or an objective lack of help offered. The cross-sectional, observational nature of these data limits causal inference. However, surveys of the perceived needs of people bereaved by suicide indicate clear unmet needs for social networks to respond proactively and empathically, and for professionals to offer immediate outreach[10]. This suggests that our findings represent gaps in support rather than a rejection or avoidance of help. Whether stigma explains the inequalities observed, perhaps by inhibiting help-seeking or offers of support, requires further research. The low rates of help-seeking after suicide attempt are particularly concerning in people bereaved by suicide given their higher risk of suicide attempt[2] and the high priority accorded to their needs for support within British suicide prevention strategies.

Results in the context of other studies

Perhaps reflecting cultural differences, our findings differ from those of a representative US sample of suicide-bereaved relatives, in which 24% had received either formal or informal support and 33% preferred to cope without assistance[34]. In a US help-seeking sample 78% reported receiving individual therapy after suicide bereavement[17]; a proportion greatly exceeding formal support use in our population-based sample. The only British study of support after suicide[35] did not state the overall proportion receiving support, but the prevalence of counselling matched that in our study. Consultation with faith leaders was double that in our sample, perhaps reflecting differing age profiles. Studies comparing groups

For peer review only

For peer review only - http://bmjopen.bmj.com/site/about/guidelines.xhtml

bereaved by suicide and other causes have only focussed on single measures of perceived social support and have, like our study, found weak or no evidence for group differences[14-16].

Strengths and limitations

This national sample represents the largest and most comprehensive survey of support received by people bereaved by a close contact's sudden death. It included respondents who were related and unrelated to the deceased, recognising that adverse outcomes and needs for support apply regardless of kinship[2]. In conducting specific group comparisons we were able to ascertain that reduced receipt of informal support was attributable to suicide bereavement rather than unnatural losses more widely. Results were robust to sensitivity analysis, and use of a precise sampling frame allowed us to be clear about the limits of generalisability. The possibility of selection bias through sampling from HEIs, and the pronounced male non-response bias limit generalisability beyond highly-educated female groups. The limited age-range sampled restricts generalisability beyond young adults. Without a denominator we were unable to present a response rate, but assume that the majority of non-responders were non-bereaved. It was possible that those worst affected had biased recall of support received and its value. Our multivariable models included pre-bereavement depression as a potential confounding variable but did not account for pre-bereavement anxiety or other mental disorders. If those are differentially elevated prior to suicide bereavement, as shown in previous studies[1], stigma associated with mental illness and/or poor experiences of services might influence receipt of support in this group. Models for two secondary outcomes (exclusive use of formal support; help-seeking for attempted suicide post-bereavement) lacked sufficient power due to group sizes <466, and larger studies are needed to investigate these hypothesised associations. Despite testing for an interaction with gender, we acknowledge such tests' limited statistical power. Given gender differences in help-seeking

For peer review only - http://bmjopen.bmj.com/site/about/guidelines.xhtml

for mental illness[22], particularly in relation to informal support[23], it would have been desirable to have conducted gender-specific analyses but this was not possible due to the low numbers of men responding.

Policy implications

The quarter of our suicide-bereaved sample who received no support represent failed implementation of UK suicide prevention strategies[4-6]. This group was distinct from the 1% who stated that they preferred to cope without assistance. The inequities in informal support we identified for people bereaved by suicide suggest a need for psychosocial interventions to address social avoidance and stigmatising attitudes within social networks. Public education to raise awareness of the vulnerabilities of people bereaved by suicide, the range of support available[36], and advice on how to support them[36;37] could encourage social networks to respond more readily after suicide loss. This, along with interventions to address self-stigma, might also encourage the bereaved to seek help by reinforcing the idea that they are worthy of support. Current UK developments in national systems of early outreach after suicide[38] will address the identified delays in support, particularly at a stage when motivation and awareness is low[10].

Further research

Research is needed to explore the influence of stigma on willingness to seek help after suicide bereavement, and on others' readiness to offer support. Thematic analysis of our qualitative survey data will permit a more nuanced understanding of this. Studies that deepen our understanding of help-seeking preferences after suicide attempt in people bereaved by suicide might help address risk of re-attempt. Expanding the limited evidence base for interventions after suicide bereavement[7] is important, as is investigating the potential for adverse psychological effects of early[39] and peer support[40] interventions.

For peer review only - http://bmjopen.bmj.com/site/about/guidelines.xhtml

Conclusions

Our study demonstrated clear inequities in the support received by people bereaved by the suicide of a close contact, manifested in delayed receipt of support and a lesser likelihood of receiving support from family and friends. It is concerning that two-thirds of a group featuring so prominently in UK suicide prevention strategies receive no formal support, and that a quarter receive no support at all. Those responsible for implementing suicide prevention strategies should commission lay guidance on how to support someone bereaved by suicide, and improve national systems of immediate outreach after suicide loss.

Acknowledgements:

We would like to thank all the HEIs from England, Wales, Northern Ireland, and Scotland that consented to participate, listed below, and all the bereaved individuals who took time to respond to the online survey.

Participating HEIs: Bishop Grosseteste University College Lincoln; Bournemouth University; Central School of Speech and Drama; City University; Cranfield University; Courtauld Institute; De Montfort University; University of Greenwich; King's College London; Liverpool Institute for Performing Arts; Liverpool John Moores University; London Metropolitan University; Norwich University College of the Arts; Royal Veterinary College; School of Oriental and African Studies; St George's, University of London; Staffordshire University; Trinity Laban Conservatoire of Music and Dance; UCL; University Campus Suffolk; University of Bedfordshire; University of Chester; University of Cumbria; University of Leeds; University of Liverpool; University of Oxford; University of Southampton; University of Worcester; University of Westminster; Queen Margaret University; Heriot-Watt University; Scottish Agricultural College; University of Dundee;

For peer review only

For peer review only - http://bmjopen.bmj.com/site/about/guidelines.xhtml

Cardiff University; Cardiff Metropolitan University (formerly University of Wales Institute Cardiff); Queen's University Belfast; University of Ulster.

Contributors: AP, DO, and MK conceived and designed the study. AP, DO, MK, PM and LS contributed to questionnaire design and recruitment strategy. AP recruited participants, managed the survey, and collected data. AP, KR and LM cleaned data. AP, KR, LM and DO conducted data analysis. AP, KR, PM, LS, LM, MK, and DO interpreted data, contributed to writing of the report and approved the final version before submission. AP conducted the literature search. AP had full access to all the data in the study and takes responsibility for the integrity of the data and the accuracy of the data analysis. No participant is identifiable from the analysis or study report. AP is the guarantor.

Ethical approval: This project was independently peer reviewed by the Medical Research Council (MRC). Ethical approval was granted by the UCL Research Ethics Committee in 2010 (reference number: 1975/002).

Funding: This project was funded by a MRC Population Health Scientist Fellowship to AP (G0802441). The MRC had no role in design and conduct of the study; collection, management, analysis, and interpretation of the data; or preparation, review, and approval of the manuscript. The views expressed are those of the authors and not necessarily those of the Medical Research Council.

Transparency: The lead author (AP) affirms that the manuscript is an honest, accurate, and transparent account of the study being reported; that no important aspects of the study have been omitted; and that any discrepancies from the study as planned have been explained.

Data sharing: Informed consent for collecting patient-level data was obtained on the basis that data would be anonymised, stored in accordance with the Data Protection Act 1998, only

For peer review only

For peer review only - http://bmjopen.bmj.com/site/about/guidelines.xhtml

used for the purposes of the study, and not transferred to an organisation outside UCL. Requests to collaborate on analysis of anonymised data with low risk of identification should be made by contacting the corresponding author.

Competing interests: All authors have completed the ICMJE uniform disclosure form at www.icmje.org/coi_disclosure.pdf and declare: financial support from the Medical Research Council for the submitted work; no financial relationships with any organisations that might have an interest in the submitted work in the previous three years; no other relationships or activities that could appear to have influenced the submitted work.

Copyright: The Corresponding Author has the right to grant on behalf of all authors and does grant on behalf of all authors, an exclusive licence on a worldwide basis to the BMJ Publishing Group Ltd to permit this article (if accepted) to be published in BMJ editions and any other BMJPGL products and sublicences such use and exploit all subsidiary rights, as set out in their licence.

Supplementary files:

Figure 1: Participant flow

Research checklist: STROBE statement

Supplementary table: Estimates of the relationship between support outcomes and bereavement exposure (suicide *versus* non-suicide sudden death)

For peer review only

Table 1: Characteristics of participants by type of bereavement exposure

Participants bereaved by:	sudden natural death (n=2106)	sudden unnatural death (n=712)	suicide (n=614)	Total (n=3,432)	p-value [a]
**Socio-demographic characteristics**					
**Gender †**					
Female n (%)	1709 (81)	576 (81)	499 (81)	2784 (81)	0·955
Missing n (%)	1 (<1)	0 (0)	0 (0)	1 (<1)	
**Age of participant †** mean (SD)	24·9 (6·3)	25·2 (6·3)	25·2 (6·0)	25·0 (6·3)	0·069
**Self-defined ethnicity**					
white n (%)	1877 (89)	645 (91)	562 (92)	3084 (90)	0·102
missing n (%)	1 (<1)	2 (<1)	0 (0)	3 (<1)	
**Socio-economic status [b] †**					
social classes 1.1 & 1.2 n (%)	603 (29)	224 (32)	176 (29)	1003 (29)	0·604
social class 2 n (%)	684 (33)	234 (33)	204 (33)	1122 (33)	
social class 3 n (%)	259 (12)	77 (11)	68 (11)	404 (12)	
social class 4 n (%)	90 (4)	34 (5)	32 (5)	156 (5)	
social classes 5,6,7 & 9 n (%)	409 (19)	115 (16)	113 (18)	638 (19)	
missing n (%)	61 (3)	27 (4)	21 (3)	109 (3)	
**Educational status**					
attained up to 2° school leaving qualification n (%)	964 (46)	286 (40)	255 (42)	1505 (44)	**0·035**
attained degree or above n (%)	1136 (54)	424 (60)	359 (59)	1919 (56)	
missing n (%)	6 (<1)	2 (<1)	0 (0)	8 (<1)	

For peer review only - http://bmjopen.bmj.com/site/about/guidelines.xhtml

Participants bereaved by:	sudden natural death (n=2106)	sudden unnatural death (n=712)	suicide (n=614)	Total (n=3,432)	p-value [a]
**Student status**					
student n (%)	1797 (85)	613 (86)	526 (86)	2936 (86)	0·905
staff n (%)	253 (12)	78 (11)	68 (11)	399 (12)	
both n (%)	55 (3)	21 (3)	20 (3)	96 (3)	
missing n (%)	1 (<1)	0 (0)	0 (0)	1 (<1)	
**Measure of social support [c]**					
no lack of perceived social support n (%)	1234 (59)	411 (58)	345 (56)	1990 (58)	0·297
moderate lack of perceived social support n (%)	549 (26)	197 (28)	168 (27)	914 (27)	
severe lack of perceived social support n (%)	323 (15)	102 (14)	100 (16)	525 (15)	
missing n (%)	0 (0)	2 (<1)	1 (<1)	3 (<1)	
**Clinical characteristics**					
**Personality disorder screen positive [d]**					
Yes n (%)	743 (35)	227 (32)	225 (37)	1195 (35)	0·071
missing n (%)	131 (6)	31 (4)	33 (5)	195 (6)	
**Family history of psychiatric problems**					
Yes n (%)	1243 (59)	434 (61)	412 (67)	2089 (61)	**0·005**
missing n (%)	153 (7)	41 (6)	39 (6)	233 (7)	
**Other family history of suicide †**					
Yes n (%)	123 (6)	41 (6)	53 (7)	217 (6)	0·071
missing n (%)	158 (8)	43 (6)	40 (7)	241 (7)	

For peer review only - http://bmjopen.bmj.com/site/about/guidelines.xhtml

Participants bereaved by:	sudden natural death (n=2106)	sudden unnatural death (n=712)	suicide (n=614)	Total (n=3,432)	p-value [a]
**Pre-loss non-suicidal self-harm & suicide attempt †**					
Yes n (%)	434 (21)	134 (19)	150 (24)	718 (21)	0·050
missing n (%)	157 (8)	41 (6)	41 (7)	239 (7)	
**Pre-loss depression †**					
Yes n (%)	370 (18)	129 (18)	143 (23)	642 (19)	**0·015**
missing n (%)	85 (4)	21 (3)	24 (4)	130 (4)	
**Characteristics of the bereavement**					
**Kinship to the deceased †**					
blood relative n (%)	1786 (85)	351 (49)	296 (48)	2433 (71)	**<0·001**
unrelated n (%)	313 (15)	356 (50)	317 (52)	980 (29)	
missing n (%)	7 (<1)	5 (1)	1 (<1)	13 (<1)	
**Age of the deceased**: mean (SD)	55.1 (21.5)	31·0 (17·4)	31·9 (15·2)	45·9 (22·8)	**<0·001**
**Time since bereavement †** : mean (SD)	4·8 (5·3)	5·3 (5·4)	5·1 (5·0)	5·0 (5·3)	0·140
**Bereavement in last 2 years**					
Yes n (%)	707 (34)	186 (26)	168 (27)	1061 (31)	**<0·001**
No n (%)	1399 (67)	526 (74)	446 (73)	2371 (69)	
**GEQ stigma subscale score** *mean (SD)*	11·9 (3·8)	12·3 (4.0)	14·0 (4·3)	12·3 (4·0)	**<0·001**

For peer review only - http://bmjopen.bmj.com/site/about/guidelines.xhtml

Participants bereaved by:	sudden natural death (n=2106)	sudden unnatural death (n=712)	suicide (n=614)	Total (n=3,432)	p-value [a]
**Time point rated as worst stage after the loss**					
within a week (%)	560 (25)	156 (22)	148 (24)	824 (24)	0.112
up to a month n (%)	330 (16)	92 (13)	81 (13)	503 (15)	
up to 6 months n (%)	330 (16)	122 (17)	112 (18)	564 (16)	
up to a year n (%)	359 (17)	147 (21)	101 (17)	607 (18)	
up to 3 years n (%)	216 (10)	80 (11)	69 (11)	365 (11)	
over 3 years n (%)	181 (9)	62 (9)	59 (10)	302 (9)	
missing n (%)	170 (8)	53 (8)	44 (7)	267 (8)	
**Bereavement support**					
**Any formal/informal support [e] received after bereavement**					
Yes n (%)	1573 (75)	558 (78)	441 (72)	2572 (75)	**0.031**
No n (%)	446 (21)	131 (18)	148 (24)	725 (21)	
Missing n (%)	87 (4)	23 (3)	25 (4)	135 (4)	
**Formal/informal support perceived to be valuable** (of n=2572)					
Yes n (%)	1,335 (85)	464 (83)	374 (85)	2173 (85)	0.621
No n (%)	216 (14)	85 (15)	59 (13)	360 (14)	
Missing n (%)	22 (1)	9 (2)	8 (2)	39 (2)	
**Type of formal/informal support received** (of n=2572)					
Formal only n (%)	217 (14)	76 (14)	68 (15)	361 (14)	0·922
Informal only n (%)	796 (51)	286 (51)	220 (50)	1302 (51)	
Both formal and informal n (%)	560 (36)	196 (35)	153 (35)	909 (35)	

For peer review only - http://bmjopen.bmj.com/site/about/guidelines.xhtml

Participants bereaved by:	sudden natural death (n=2106)	sudden unnatural death (n=712)	suicide (n=614)	Total (n=3,432)	p-value [a]
**Point at which any valuable support received after loss**					
within a day n(%)	623 (30)	234 (33)	150 (24)	1007 (29)	**0·001**
within a week n(%)	290 (14)	72 (10)	69 (11)	431 (13)	
within a month n(%)	154 (7)	50 (7)	44 (7)	248 (7)	
within 6 months n(%)	117 (6)	35 (5)	46 (8)	198 (6)	
within a year n(%)	58 (3)	31 (4)	15 (2)	104 (3)	
over a year n(%)	124 (6)	49 (7)	58 (10)	231 (7)	
at no point n(%)	632 (30)	211 (30)	198 (32)	1041 (30)	
missing n (%)	108 (5)	30 (4)	34 (6)	172 (5)	
**Whether help sought after self-harm post-bereavement [f]**					
Yes n (%)	42/112 (38)	8/42 (19)	19/56 (34)	69/210 (33)	0.093
No n (%)	70/112 (63)	34/42 (81)	37/56 (66)	141/210 (67)	

† = pre-specified confounding variable used in adjusted models
[a] significance threshold of p=0·05; not adjusted for multiple testing
[b] socio-economic status using the 5 categories from UK Office for National Statistics
[c] measure of social support from Adult Psychiatric Morbidity Survey (25)
[d] SAPAS-SR screen for personality disorder (33)
[e] excluding self-help
[f] in sub-sample of n=210 who had made a suicide attempt since the index bereavement

For peer review only - http://bmjopen.bmj.com/site/about/guidelines.xhtml

Table 2: Specific type of support used after bereavement

Participants bereaved by:	sudden natural death (n=2106) n (% of exposure group)	sudden unnatural death (n=712) n (% of exposure group)	suicide (n=614) n (% of exposure group)	Total (n=3,432) n (% of total sample)
**Specific bereavement support reported [a]**				
**Formal support**				
health services (doctor, nurse, therapist, counsellor)	283 (13)	86 (12)	83 (14)	452 (13)
social services	0 (0)	0(0)	1(<1)	1 (<1)
private counsellor or therapist	171 (8)	78 (11)	73 (12)	322 (9)
voluntary sector services (helpline, counsellor)	120 (6)	53 (7)	51 (8)	224 (7)
police officers	77 (4)	102 (14)	45 (7)	224 (7)
funeral directors	359 (17)	85 (12)	51 (8)	495 (14)
coroners' officers	130 (6)	51 (7)	35 (6)	216 (6)
school teachers or school counselling services	28 (1)	11 (2)	9 (2)	48 (1)
college tutor or college counselling services	34 (2)	11 (2)	19 (3)	64 (2)
line manager or employee counselling services	5 (<1)	3 (<1)	1 (<1)	9 (<1)
Subtotal formal support	1207 (57)	480 (67)	368 (60)	2055 (60)
**Informal support**				
friends and family	1349 (64)	481 (68)	370 (60)	2200 (64)
spiritual/religious advisors	40 (2)	10 (1)	10 (2)	60 (2)
complementary and alternative medicine (CAM)	1 (<1)	0 (0)	0 (0)	1 (<1)
Subtotal informal support	1390 (66)	491 (69)	380 (62)	2261 (66)
Subtotal any formal or informal support	1573 (75)	558 (78)	441 (72)	2572 (75)

For peer review only - http://bmjopen.bmj.com/site/about/guidelines.xhtml

Participants bereaved by:	sudden natural death (n=2106) n (% of exposure group)	sudden unnatural death (n=712) n (% of exposure group)	suicide (n=614) n (% of exposure group)	Total (n=3,432) n (% of total sample)
**Other**				
self-help (website, book, leaflet)	208 (10)	61 (9)	79 (13)	348 (10)
Specific source not specified	23 (1)	7 (1)	6 (1)	36 (1)
Other (not classified as above)[b]	3 (<1)	2 (<1)	1 (<1)	6 (<1)
Subtotal Other	234 (11)	70 (10)	86 (14)	390 (11)
**None**				
Chose to handle it alone [c]	15 (<1)	4 (1)	1 (<1)	20 (1)
No help received [d] n(%)	428 (20)	129 (18)	141 (23)	698 (20)
**Specific support sought following any self-harm post-bereavement** [e]				
none	70 (63)	34 (81)	37 (66)	141 (67)
friend	18 (16)	2 (5)	8 (14)	28 (13)
family member	13 (12)	3 (7)	7 (13)	23 (11)
general practitioner (GP)	25 (22)	5 (12)	12 (21)	42 (20)
hospital professionals	10 (9)	1 (2)	5 (9)	16 (8)
counsellor	9 (8)	1 (2)	4 (7)	13 (6)
mental health team member	2 (2)	0 (0)	3 (5)	5 (2)
voluntary sector organisation	1 (1)	0 (0)	0 (0)	1 (<1)
school/college teaching staff	2 (2)	0 (0)	0 (0)	1 (<1)

For peer review only - http://bmjopen.bmj.com/site/about/guidelines.xhtml

[a] categories not mutually exclusive
[b] category included organisations such as the diplomatic service, shipping services (for repatriating the body), and employees at the deceased's bank.
[c] 16/20 people in this category also endorsed other sources of formal or informal support
[d] category excluded those who had used self-help and those who indicated they had chosen to handle the bereavement alone
[e] in the n=210 individuals who had attempted suicide post-bereavement; categories not mutually exclusive

For peer review only

For peer review only - http://bmjopen.bmj.com/site/about/guidelines.xhtml

Table 3: Estimates of the relationship between support outcomes and bereavement exposure (suicide *versus* sudden natural death)

Exposure group	Sudden natural death (n = 2106)		Sudden unnatural death (n = 712)					Suicide (n = 614)				
**Primary outcomes**	**Prevalence n (%)**	**Odds ratio** (reference)	**Prevalence n (%)**	**Unadjusted odds ratio (95% CI)**	***p* value***	**Adjusted[a] odds ratio (95% CI)**	***p* value***	**Prevalence n (%)**	**Unadjusted odds ratio (95% CI)**	***p* value***	**Adjusted[a] odds ratio (95% CI)**	***p* value***
receipt of formal support post-bereavement	776 (37)	1	272 (38)	1.05 (0.88-1.27)	0.548	**1.28† (1.05-1.56)**	**0.015**	221 (36)	0.97 (0.80-1.18)	0.753	1.17 (0.94-1.44)	0.155
receipt of informal support post-bereavement	1396 (66)	1	491 (69)	1.13 (0.92-1.38)	0.257	1.06 (0.86-1.33)	0.553	389 (63)	0.83 (0.68-1.02)	0.083	**0.79† (0.64-0.98)**	**0.038**
**Secondary outcomes**												
no support post-bereavement [b]	428 (20)	1	129 (18)	0.83 (0.66-1.05)	0.122	0.83 (0.65-1.07)	0.149	141 (23)	1.21 (0.97-1.52)	0.097	1.21 (0.95-1.55)	0.119
immediate receipt of support (<1 week)	913 (43)	1	306 (43)	0.97 (0.81-1.17)	0.747	0.96 (0.79-1.17)	0.660	219 (36)	**0.74 (0.60-0.90)**	**0.002**	**0.73† (0.59-0.90)**	**0.003**
delayed receipt of valuable support (>6 months)	814 (39)	1	291 (41)	1.05 (0.88-1.27)	0.575	1.10 (0.90-1.35)	0.359	271 (44)	1.26 (1.04-1.53)	0.020	**1.33† (1.08-1.64)**	**0.008**
use of formal support exclusively [c]	217/1573 (14)	1	76/558 (14)	0.98 (0.72-1.33)	0.888	1.11 (0.80-1.54)	0.516	68/441 (15)	1.12 (0.82-1.55)	0.454	1.26 (0.90-1.76)	0.183
help sought for post-bereavement self-harm [d]	42/112 (38)	1	8/42 (19)	0.37 (0.15-0.95)	0.038	0.43 (0.16-1.13)	0.086	19/56 (34)	0.82 (0.41-1.65)	0.579	0.98 (0.44-2.17)	0.953

[a] adjusted for age, gender, socio-economic status, pre-loss depression, pre-loss suicidal and non-suicidal self-harm, other family history of suicide (excluding index bereavement), time since bereavement, and kinship to the deceased.

[b] outcome excluded those who solely endorsed that they chose to handle the bereavement alone

[c] in sub-set of n=2,572 receiving support after bereavement

[d] in sub-set of n=210 who had attempted suicide post-bereavement
* significance threshold of p=0·05 for primary outcomes and p=0·01 for secondary outcomes
† association no longer significant when stigma added to final adjusted model

For peer review only

Table 4: Estimates of the relationship between support outcomes and bereavement exposure (suicide *versus* sudden unnatural death)

**Exposure group**	**Sudden unnatural death (n = 712)**	**Suicide (n = 614)**			
**Primary outcomes**	**Odds ratio** (reference)	**Unadjusted odds ratio (95% CI)**	***p* value***	**Adjusted[a] odds ratio (95% CI)**	***p* value***
receipt of formal support post-bereavement	1	0.92 (0.72-1.16)	0.462	0.91 (0.72-1.16)	0.437
receipt of informal support post-bereavement	**1**	**0.74 (0.58-0.95)**	**0.020**	**0.74† (0.58-0.96)**	**0.022**
**Secondary outcomes**					
no support post-bereavement [b]	1	1.46 (0.95-1.52)	0.010	1.46 (1.09-1.95)	0.011
immediate receipt of support (<1 week)	1	0.76 (0.60-96)	0.021	0.76 (0.60-0.97)	0.025
delayed receipt of valuable support (>6 months)	1	1.19 (0.94-1.51)	0.139	1.21 (0.95-1.54)	0.120
use of formal support exclusively [c]	1	1.16 (0.79-1.70)	0.463	1.13 (0.77-1.67)	0.542
help sought for post-bereavement self-harm [d]	1	2.18 (0.79-5-98)	0.131	2.28 (0.78-6.68)	0.132

Footnotes as per Table 3

For peer review only - http://bmjopen.bmj.com/site/about/guidelines.xhtml

Reference List

(1) Pitman A, Osborn DPJ, King MB, Erlangsen A. Effects of suicide bereavement on mental health and suicide risk. *Lancet Psychiatry* 2014;1:86-94.

(2) Pitman AL, Osborn DPJ, Rantell K, King MB. Bereavement by suicide as a risk factor for suicide attempt: a cross-sectional national UK-wide study of 3432 young bereaved adults. *BMJ Open* 2016;6.

(3) U.S.Department of Health and Human Services (HHS) Office of the Surgeon General and National Action Alliance for Suicide Prevention. National Strategy for Suicide Prevention: Goals and Objectives for Action. 2012. Washington, DC: HHS.

(4) Department of Health. Preventing suicide in England: A cross-government outcomes strategy to save lives. 17680, 1-57. 2012. published online 10 September 2012.

(5) Department of Health Social Services and Public Safety. Protect Life - A Shared Vision. The Northern Ireland Suicide Prevention Strategy 2012-2014. 2012.

(6) Welsh Government. Talk To Me 2 - Suicide and Self Harm Prevention Strategy for Wales 2015-2020 . 2015.

(7) McDaid C, Trowman R, Golder S, Hawton K, Sowden A. Interventions for people bereaved through suicide: systematic review. *Br J Psychiatry* 2008;193:438-443.

(8) Diekstra RFW. Suicide and the attempted suicide: An international perspective. *Acta Psychiatr Scand* 1989;80:1-24.

(9) Cvinar JG. Do Suicide Survivors Suffer Social Stigma: A Review of the Literature. *Perspect Psychiatr Care* 2005;41:14-21.

For peer review only - http://bmjopen.bmj.com/site/about/guidelines.xhtml

(10) Dyregrov K. What Do We Know About Needs for Help After Suicide in Different Parts of the World? *Crisis* 2011;32:310-318.

(11) Pitman AL, Osborn DPJ, Rantell K, King MB. The stigma perceived by people bereaved by suicide and other sudden deaths: A cross-sectional UK study of 3432 bereaved adults. *J Psychosom Res* 2016;87:22-29.

(12) Chapple A, Ziebland S, Hawton K. Taboo and the different death? Perceptions of those bereaved by suicide or other traumatic death. *Sociol Health Illn* 2015;37:610-625.

(13) Rusch N, Zlati A, Black G, Thornicroft G. Does the stigma of mental illness contribute to suicidality? *Br J Psychiatry* 2014;205:257-259.

(14) Farberow NL, Gallagher-Thompson D, Gilewski M, Thompson L. The role of social supports in the bereavement process of surviving spouses of suicide and natural deaths. *Suicide Life Threat Behav* 1992;22:107-124.

(15) Seguin M, Lesage A, Kiely MC. Parental bereavement after suicide and accident: a comparative study. *Suicide Life Threat Behav* 1995;25:489-492.

(16) Range L.M., Niss N.M. Long-term bereavement from suicide, homicide, accidents and natural deaths. *Death Stud* 1990;14:423-433.

(17) McMenamy J.M., Jordan JR, Mitchell AM. What do Suicide Survivors Tell Us They Need? Results of a Pilot Study. *Suicide Life Threat Behav* 2008;38:375-389.

(18) Bolton JM, Au W, Leslie WD et al. Parents bereaved by offspring suicide: a population-based longitudinal case-control study. *JAMA Psychiatry* 2013;70:158-167.

For peer review only

(19) Kuramoto S, Runeson B, Stuart EA, Lichtenstein P, Wilcox HC. Time to hospitalization for suicide attempt by the timing of parental suicide during offspring early development. *JAMA Psychiatry* 2013;70:149-157.

(20) Kuramoto SJ, Stuart EA, Runeson B, Lichtenstein P, Langstrom N, Wilcox HC. Maternal or Paternal Suicide and Offspring's Psychiatric and Suicide-Attempt Hospitalization Risk. *Pediatrics* 2010;126:e1026-e1032.

(21) Wilcox H.C., Kuramoto S.J., Lichtenstein P, Langstrom N, Brent D.A., Runeson B. Psychiatric Morbidity, Violent Crime, and Suicide Among Children and Adolescents Exposed to Parental Death. *J Am Acad Child Adolesc Psychiatry* 2010;49:514-523.

(22) Pitman A, Krysinska K, Osborn D, King M. Suicide in young men. *Lancet* 2012;379:2383-2392.

(23) Brown J, Evans-Lacko S, Aschan L, Henderson M, Hatch S, Hotopf M. Seeking informal and formal help for mental health problems in the community: a secondary analysis from a psychiatric morbidity survey in South London. *BMC Psychiatry* 2014;14:1-15.

(24) Pitman AL, Osborn DPJ, King MB. The use of internet-mediated cross-sectional studies in mental health research. *BJPsych Advances* 2015;21:175-184.

(25) McManus S, Meltzer H, Brugha T, Bebbington P, Jenkins R. Adult psychiatric morbidity in England, 2007. Results of a household survey. 1-141. 2009. Leeds, The Health and Social Care Information Centre; National Centre for Social Research; A survey carried out for The NHS Information Centre for health and social care by the National Centre for Social Research.

For peer review only

For peer review only - http://bmjopen.bmj.com/site/about/guidelines.xhtml

(26) Bebbington PE, Minot S, Cooper C et al. Suicidal ideation, self-harm and attempted suicide: Results from the British psychiatric morbidity survey 2000. *European Psychiatry* 2010;25:427-431.

(27) Robins LN, Wing JK, Wittchen H-U et al. The Composite International Diagnostic Interview. An epidemiologic instrument for use in conjunction with different diagnostic systems and in different cultures. *Arch Gen Psychiatry* 1988;45:1069-1077.

(28) Bailley SE, Dunham K, Kral MJ. Factor structure of The Grief Experience Questionnaire (GEQ). *Death Stud* 2000;24:721-738.

(29) Bruffaerts R, Demyttenaere K, Hwang I et al. Treatment of suicidal people around the world. *Br J Psychiatry* 2011;199:64-70.

(30) Stevenson FA, Britten N, Barry CA, Bradley CP, Barber N. Self-treatment and its discussion in medical consultations: how is medical pluralism managed in practice? *Soc Sci Med* 2003;57:513-527.

(31) Stroebe M, Schut H, Stroebe W. Health outcomes of bereavement. *Lancet* 2007;370:1960-1973.

(32) *Stata Statistical Software: Release 12* College Station, TX: StataCorp LP: StataCorp LP; 2011.

(33) Germans S, Van Heck GL, Moran P, Hodiamont PPG. The Self-report Standardized Assessment of Personality-abbreviated Scale: Preliminary results of a brief screening test for personality disorders. *Personality* 2008;2:70-76.

For peer review only

For peer review only - http://bmjopen.bmj.com/site/about/guidelines.xhtml

(34) Provini C, Everett JR, Pfeffer C.R. Adults mourning suicide: self-reported concerns about bereavement, needs for assistance, and help-seeking behavior. *Death Stud* 2000;24:1-19.

(35) Harwood D., Hawton K., Hope T., Jacoby R. The grief experiences and needs of bereaved relatives and friends of older people dying through suicide: a descriptive and case-control study. *J Affect Disord* 2002;72:185-194.

(36) Public Health England, National Suicide Prevention Alliance. Help is at Hand: Support after someone may have died by suicide. 2015. London, HMSO.

(37) Carson J Spencer Foundation, National Action Alliance for Suicide Prevention, American Association of Suicidology. A Manager's Guide to Suicide Postvention in the Workplace: 10 Action Steps for Dealing with the Aftermath of Suicide. 2013. Denver, CO., Carson J Spencer Foundation.

(38) Department of Health. Preventing suicide in England: Two years on. Second annual report on the cross-government outcomes strategy to save lives. SCLGCP/ MHED / 11160. 2015.

(39) Wessely S, Deahl M. Psychological debriefing is a waste of time. *Br J Psychiatry* 2003;183:12-14.

(40) de Groot M, Kollen BJ. Course of bereavement over 8-10 years in first degree relatives and spouses of people who committed suicide: longitudinal community based cohort study. *BMJ* 2013;347.

For peer review only - http://bmjopen.bmj.com/site/about/guidelines.xhtml

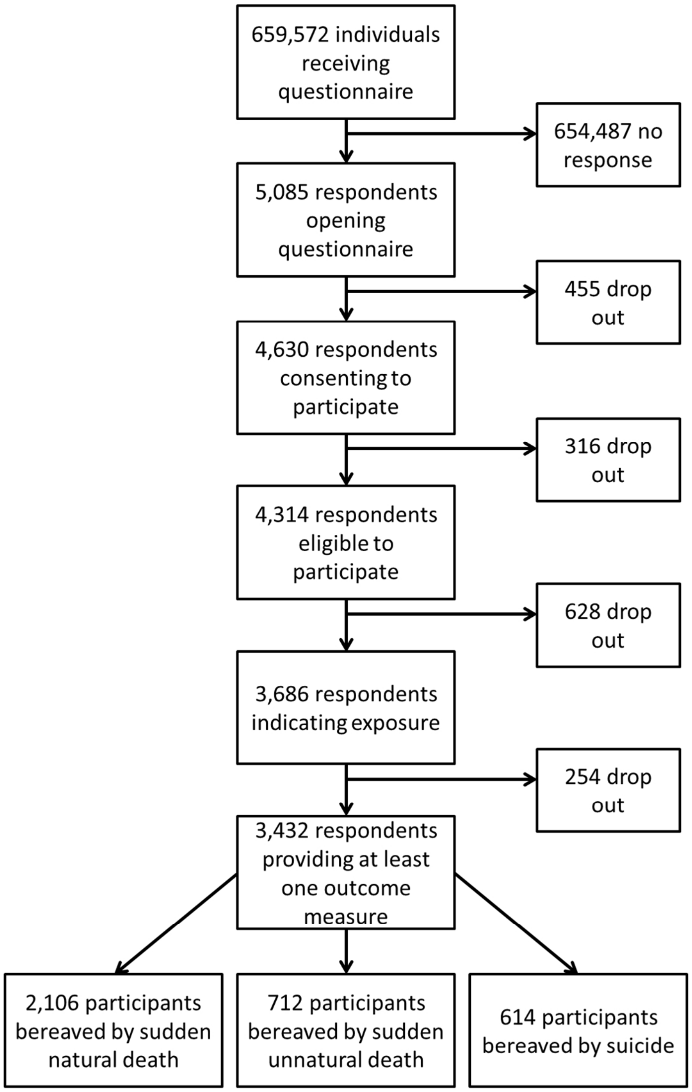

111x159mm (300 x 300 DPI)

Supplementary Table: Estimates of the relationship between support outcomes and bereavement exposure (suicide *versus* non-suicide sudden death)

Exposure group	Non-suicide sudden death (n=2818)		Suicide (n = 614)				
**Primary outcomes**	**Prevalence n (%)**	**Odds ratio** (reference)	**Prevalence n (%)**	**Unadjusted odds ratio (95% CI)**	***p* value***	**Adjusted[a] odds ratio (95% CI)**	***p* value***
receipt of formal support post-bereavement	1048 (37)	1	221 (36)	0.96 (0.79-1.16)	0.644	1.08 (0.88-1.31)	0.472
receipt of informal support post-bereavement	1887 (67)	1	389 (63)	0.82 (0.67-0.99)	0.042	**0.78† (0.64-0.96)**	**0.018**
**Secondary outcomes**							
no support post-bereavement [b]	557 (20)	1	141 (23)	1.31 (1.05-1.62)	0.016	1.32 (1.06-1.67)	0.014
immediate receipt of support (<1 week)	1219 (43)	1	219 (36)	**0.74 (0.61-0.90)**	**0.002**	**0.74† (0.61-0.90)**	**0.003**
delayed receipt of valuable support (>6 months)	1105 (39)	1	271 (44)	1.24 (1.02-1.49)	0.028	1.28 (1.05-1.56)	0.017
use of formal support exclusively [c]	293/2131 (14)	1	68/441 (15)	1.18 (0.88-1.59)	0.264	1.27 (0.93-1.72)	0.130
help sought for post-bereavement self-harm [d]	50/154 (32)	1	19/56 (34)	1.02 (0.52-2.02)	0.949	1.22 (0.57-2.61)	0.612

[a] adjusted for age, gender, socio-economic status, pre-loss depression, pre-loss suicidal and non-suicidal self-harm, other family history of suicide (excluding index bereavement), time since bereavement, and kinship to the deceased.
[b] outcome excluded those who solely endorsed that they chose to handle the bereavement alone
[c] in sub-set of n=2,572 receiving support after bereavement
[d] in sub-set of n=210 who had attempted suicide post-bereavement
* significance threshold of p=0·05 for primary outcomes and p=0·01 for secondary outcomes
† association no longer significant when stigma added to final adjusted model

STROBE checklist for cross-sectional study of support received after bereavement by suicide and other sudden deaths (UCL Bereavement Study)

Corresponding author: Dr Alexandra Pitman 27 September 2016

Checklist of items that should be included in reports of ***cross-sectional studies***: green denotes page number http://www.strobe-statement.org/index.php?id=available-checklists

	Item No	Recommendation
**Title and abstract**	1	**(*a*) Indicate the study's design with a commonly used term in the title or the abstract:** abstract/title indicates that we conducted a national cross-sectional study
**Page 1 & 2-3**		**(*b*) Provide in the abstract an informative and balanced summary of what was done and what was found:** abstract outlines our hypothesis, exposures and outcomes, and adjusted odds ratio for the associations hypothesised
**Introduction**		
Background/rationale **Page 4**	2	**Explain the scientific background and rationale for the investigation being reported:** Our introduction outlines the policy context, including key research references, and highlights the lack of evidence to support current suicide prevention strategy.
Objectives **Page 3 & 4**	3	**State specific objectives, including any prespecified hypotheses:** Objectives and primary hypothesis stated in the Abstract and Introduction. Our objective was to conduct a population-based survey comparing the support received by people bereaved by different modes of sudden death and to test specific hypotheses regarding inequalities in support received by people bereaved by suicide.
**Methods**		
Study design **Page 5-6**	4	**Present key elements of study design early in the paper:** Cross-sectional survey stated in Methods.
Setting **Page 5-6**	5	**Describe the setting, locations, and relevant dates, including periods of recruitment, exposure, follow-up, and data collection**: Describes emailing individuals at 37 HEIs in 2010 for cross-sectional data collection. Acknowledgement section details the locations of diverse participating HEIs.
Participants **Page 5-6**	6	**Give the eligibility criteria, and the sources and methods of selection of participants:** Eligibility criteria described as: people aged 18-40 who had experienced sudden bereavement of a close friend or relative after ten years of age.
Variables **Page 7-8**	7	**Clearly define all outcomes, exposures, predictors, potential confounders, and effect modifiers. Give diagnostic criteria, if applicable.** All outcomes described, denoting how these were derived. Exposure clearly defined. Eight pre-specified confounding variables defined and justified. Kinship defined as a potential effect modifier.
Data sources/ measurement **Page 5 &7-8**	8*	**For each variable of interest, give sources of data and details of methods of assessment (measurement). Describe comparability of assessment methods if there is more than one group:** Questionnaire development and content described. Same instrument used for all exposure groups.
Bias **Page 5-6**	9	**Describe any efforts to address potential sources of bias:** We describe how we followed-up non-responding HEIs to ensure a diverse representation of HEIs, and how we masked participants to the study hypothesis. We also describe a decision to use two-tailed analysis to reduce inductive bias.
Study size	10	**Explain how the study size was arrived at:** We based our sample size calculation on the primary outcome of a separate study to describe the association between suicide

For peer review only - http://bmjopen.bmj.com/site/about/guidelines.xhtml

**Page 6**		bereavement and suicide attempt (the rarest outcome), to detect a doubling of the UK community prevalence of lifetime suicide attempt (6·5%) in young adult samples.
Quantitative variables<br>**Page 5-8**	11	**Explain how quantitative variables were handled in the analyses. If applicable, describe which groupings were chosen and why:** Our Methods section defines the 3 exposure groups, 7 outcomes, and 8 covariates; and how each was used in the analysis.
Statistical methods<br>**Page 8-9**	12	**(*a*) Describe all statistical methods, including those used to control for confounding:** We describe our use of multivariable logistic regression, including justification of the 8 covariates used in the adjusted models.
		**(*b*) Describe any methods used to examine subgroups and interactions:** We describe how we tested for an interaction with kinship.
		**(*c*) Explain how missing data were addressed:** We explain that levels of missing data were low (<7% for model covariates, and <4% for outcomes) and so our statistician co-authors advised that we did not need to use best and worst case scenarios to impute missing values as part of our sensitivity analyses.
		**(*d*) If applicable, describe analytical methods taking account of sampling strategy:** We describe our use of a cluster variable to take into account the potential for clustering of responses at the HEI level.
		**(*e*) Describe any sensitivity analyses:** We describe sensitivity analyses that assessed the impact of simulating more stringent inclusion criteria for the sampling strategy.
**Results**		
Participants<br>**Page 9, Figure 1**	13*	**(a) Report numbers of individuals at each stage of study—eg numbers potentially eligible, examined for eligibility, confirmed eligible, included in the study, completing follow-up, and analysed:** We specify numbers of those participating, consenting, and eligible, and present the participant flow in Figure 1.
		**(b) Give reasons for non-participation at each stage:** numbers not consenting, not eligible, not indicating exposure group, and not providing at least 1 outcome measure presented in Figure 1.
		**(c) Consider use of a flow diagram**: see Figure 1
Descriptive data<br>**Page 9-10, Tables 1 & 2**	14*	**(a) Give characteristics of study participants (eg demographic, clinical, social) and information on exposures and potential confounders:** Tables 1 & 2 and text indicates descriptive characteristics by exposure group.
		**(b) Indicate number of participants with missing data for each variable of interest**: Tables 1 & 2 provide proportion of missing values for each covariate of interest by exposure group.
Outcome data<br>**Table 3**	15*	**Report numbers of outcome events or summary measures:** Table 3 presents prevalence for each outcome by exposure group.
Main results<br>**Page 10-11, Tables 3 & 4**	16	**(*a*) Give unadjusted estimates and, if applicable, confounder-adjusted estimates and their precision (eg, 95% confidence interval). Make clear which confounders were adjusted for and why they were included:** Text and Tables 3 and 4 provide unadjusted and adjusted estimates, with 95% confidence intervals and p-values.
		**(*b*) Report category boundaries when continuous variables were categorized**: N/A
		**(*c*) If relevant, consider translating estimates of relative risk into absolute risk for a meaningful time period:** N/A
Other analyses<br>**Page 11**	17	**Report other analyses done—eg analyses of subgroups and interactions, and sensitivity analyses:** We report: results of adding stigma to final models, tests for an interaction with gender, sensitivity analyses.
**Discussion**		
Key results<br>**Page 11-12**	18	**Summarise key results with reference to study objectives:** The start of our discussion summarises the principle findings in relation to our main hypothesis.
Limitations	19	**Discuss limitations of the study, taking into account sources of potential bias or**

For peer review only - http://bmjopen.bmj.com/site/about/guidelines.xhtml

**Page 12-13**		**imprecision. Discuss both direction and magnitude of any potential bias:** Our discussion summarises both the strengths and weaknesses of this study, both in comparison with other potential approaches, and other previously-used approaches. We consider the possibility of either over- or under-estimation of risks given specific potential biases.
Interpretation **Page 11-13**	20	**Give a cautious overall interpretation of results considering objectives, limitations, multiplicity of analyses, results from similar studies, and other relevant evidence:** Our discussion compares our findings to the existing literature and comments on the degree to which our findings are consistent with this, and the extent to which they contribute to policy developments in relation to provision of support after suicide bereavement.
Generalisability **Page 12-13**	21	**Discuss the generalisability (external validity) of the study results:** We explore the degree to which findings from a primarily female and highly-educated UK HEI population are generalizable, either in the UK or internationally.
**Other information**		
Funding **Page 15**	22	**Give the source of funding and the role of the funders for the present study and, if applicable, for the original study on which the present article is based:** Our footnotes identify the MRC as the funder, and the limits of their role in this study.

*Give information separately for exposed and unexposed groups.

Note: An Explanation and Elaboration article discusses each checklist item and gives methodological background and published examples of transparent reporting. The STROBE checklist is best used in conjunction with this article (freely available on the Web sites of PLoS Medicine at http://www.plosmedicine.org/, Annals of Internal Medicine at http://www.annals.org/, and Epidemiology at http://www.epidem.com/). Information on the STROBE Initiative is available at www.strobe-statement.org.

For peer review only

For peer review only - http://bmjopen.bmj.com/site/about/guidelines.xhtml
